# Intravenous injection of human umbilical cord-derived mesenchymal stem cells ameliorates not only blood glucose but also nephrotic complication of diabetic rats through autophagy-mediated anti-senescent mechanism

**DOI:** 10.1186/s13287-023-03354-z

**Published:** 2023-05-29

**Authors:** Xinyue Li, Le Guo, Jingan Chen, Haowei Liang, Yi Liu, Wei Chen, Li Zhou, Letian Shan, Hui Wang

**Affiliations:** 1 School of Pharmaceutical Sciences, Zhejiang Chinese Medical University, Hangzhou, China; 2 The First Affiliated Hospital, Zhejiang Chinese Medical University, Hangzhou, China; 3 Cell Resource Bank and Integrated Cell Preparation Center of Xiaoshan District, Hangzhou Regional Cell Preparation Center (Shangyu Biotechnology Co., Ltd), Hangzhou, China; 4 Cancer Institute of Integrated Traditional Chinese and Western Medicine, Key Laboratory of Cancer Prevention and Therapy Combining Traditional Chinese and Western Medicine of Zhejiang Province, Zhejiang Academy of Traditional Chinese Medicine, Tongde Hospital of Zhejiang Province, 234 Gucui Road, Hangzhou, 310012 Zhejiang China

**Keywords:** Human umbilical cord-derived mesenchymal stem cells, Diabetic nephropathy, Podocytes, Paracrine effect, AMPK/mTOR signaling

## Abstract

**Background:**

Diabetic nephropathy (DN) is one of the most severe complications of diabetes mellitus, which is characterized by early occurrence of albuminuria and end-stage glomerulosclerosis. Senescence and autophagy of podocytes play an important role in DN development. Human umbilical cord-derived mesenchymal stem cells (hucMSCs) have potential in the treatment of diabetes and its complications. However, the role of hucMSCs in the treatment of DN and the underlying mechanism remain unclear.

**Methods:**

In vivo, a streptozotocin-induced diabetic male Sprague Dawley rat model was established to determine the renoprotective effect of hucMSCs on DN by biochemical analysis, histopathology, and immunohistochemical staining of renal tissues. And the distribution of hucMSCs in various organs in rats within 168 h was analyzed. In vitro, CCK8 assay, wound healing assay, and β-galactosidase staining were conducted to detect the beneficial effects of hucMSCs on high glucose-induced rat podocytes. Real-time PCR and western blot assays were applied to explore the mechanism of action of hucMSCs.

**Results:**

The in vivo data revealed that hucMSCs were distributed into kidneys and significantly protected kidneys from diabetic damage. The in vitro data indicated that hucMSCs improved cell viability, wound healing, senescence of the high glucose-damaged rat podocytes through a paracrine action mode. Besides, the altered expressions of senescence-associated genes (*p16*, *p53*, and *p21*) and autophagy-associated genes (*Beclin-1*,* p62*, and *LC3*) were improved by hucMSCs. Mechanistically, hucMSCs protected high glucose-induced injury in rat podocytes by activating autophagy and attenuating senescence through the AMPK/mTOR pathway.

**Conclusions:**

In conclusion, hucMSCs might be a promising therapeutic strategy for the clinical treatment of DN-induced renal damages.

**Supplementary Information:**

The online version contains supplementary material available at 10.1186/s13287-023-03354-z.

## Background

Diabetic nephropathy (DN) is by far one of the most serious complications of diabetes and the leading cause of end-stage renal disease [1]. According to the latest statistics, 40% of people with diabetes worldwide are expected to develop DN [2]. The pathogenesis of DN is related to long-term diabetes-related metabolic and hemodynamic disturbances, the core of which remains the elevated risk factors of renal cell inflammation, fibrosis and aging caused by hyperglycemia [3]. The development of DN was reported to be closely related to the senescence of the glomerular cells, in which podocytes senescence was the initiating process of glomerulosclerosis [4, 5]. Podocytes are highly specialized, terminally differentiated epithelial cells in the glomerulus and are an important component of the glomerular filtration barrier [6, 7]. Recent researches have demonstrated that high glucose environment could directly induce premature aging of podocytes, which could lead to dysfunction or damage of podocytes [8, 9]. These injured podocytes could cause proteinuria and glomerulosclerosis, and ultimately exacerbate the progression of DN [10]. Currently, the clinical use of renin–angiotensin–aldosterone system inhibitors to lower blood pressure and the strict glycemic control are two major strategies to slow the progression of DN [11, 12]. However, these treatments still have some side effects, such as increased risks of acute kidney injury and hypoglycemia [13, 14]. Therefore, new alternative therapies with better efficacy and safety remain an urgent clinical need.

Mesenchymal stem cells (MSCs) are stromal cells with the capacity for self-renewal and multidirectional differentiation, which can be isolated from a variety of tissues, such as umbilical cords, adipose tissue, and bone marrow. [15]. In recent years, MSCs have shown powerful therapeutic potentials for diabetes and for its related complications in cellular and animal models [16]. Multiple consecutive infusions of bone marrow-derived MSCs (bMSCs) could reverse hyperglycemia in type 2 diabetic rats [17], and intravenous injections of adipose-derived MSCs (ADSCs) could protect islet cells and lower blood glucose in type 2 diabetic mice by improving insulin sensitivity and reducing inflammation [18, 19]. Some other studies have revealed potential effects of bMSCs, ADSCs, and human umbilical cord-derived MSCs (hucMSCs) on DN. An in vitro study have shown that ADSCs could protect podocytes from high glucose-induced apoptosis and injury through secretion of epithelial growth factor [20]. Intravenous injection of bMSCs was shown to ameliorate DN and prevent renal failure through secreting renal trophic factors in diabetic mice [21]. Intravenous injection of hucMSCs could prevent renal fibrosis and proteinuria by inhibiting inflammation and could protect human glomerular mesangial cells by reducing oxidative damage and apoptosis [22, 23], indicating anti-DN potential of hucMSCs. Although bMSCs, ADSCs, and hucMSCs showed anti-DN potential, the allogeneic hucMSCs would be more convenient and accessible than autologous bMSCs or ADSCs in clinical applications. Nonetheless, there is still few evidence concerning the anti-DN efficacy and mechanism of hucMSCs.

By using streptozotocin (STZ)-induced DN rat model with similar anatomical features and physiological characteristics of human beings and high glucose-induced podocytes injury model, this study aimed to investigate the efficacy and mechanism of hucMSCs against renal injury in DN. Considering the secreted soluble factors from MSCs, paracrine effect is expected to play an important role during the regeneration process [24, 25]. Therefore, hucMSCs-conditioned medium (MSC-CM) was applied to investigate the paracrine action of hucMSCs and a comparison with co-cultured hucMSCs was made. A molecular mechanism of hucMSCs was explored by focusing on the AMPK/mTOR signaling-mediated attenuation of high glucose-induced senescence in renal podocytes.

## Materials and methods

### Flow cytometry identification of hucMSCs

Characterization of the hucMSCs phenotype was performed by flow cytometry. Briefly, cells were collected in phosphate buffer saline (PBS) (Zhejiang Senrui Biotechnology, Huzhou, China) at a density of 1 × 10^6^ cells/ml and incubated with the following antibodies: P-phycoerythrin (PE)-conjugated anti-CD73, PE-conjugated anti-CD90, PE-conjugated anti-CD105, PE-conjugated anti-CD34, PE-conjugated anti-CD45, PE-conjugated anti-CD11b, PE-conjugated anti-CD19, and PE-conjugated anti-HLA-DR at 4 °C in dark for 30 min. The isotope controls used were PE-Mouse IgG1 and PE-Mouse IgG2a. The labeled cells with the above antibodies and their corresponding isotope control were separately analyzed through a multicolor flow cytometer (BD Accuri C6, NY, USA), and then the result pictures were fitted and compared.

### Animals

Specific pathogen-free and healthy male Sprague–Dawley rats (200 ± 10 g, 6 weeks, Shanghai SLAC Laboratory Animal Co. Ltd., China) were used in this study. During the experiment, animals' rooms were regularly cleaned up, and sufficient feed and water were provided twice a day. Rats were monitored for signs of disease (illness, injury, or abnormal behavior) at least once a day by animal care and veterinary staff, and the frequency of observation is increased when animals showed signs of abnormality. Rats were transferred to individual cages when their condition deteriorated to such an extent that access to water/food was compromised or that they might be harmed by other animals. Dying or dead animals were quickly removed from their cages. This study was approved by the Committee of Animal Care and Use of Zhejiang Chinese Medical University (Animal Ethics No: IACUC-20211101–13). Rats were maintained under standard feeding conditions (temperature 20 ± 2 °C; humidity 45 ~ 55%; 12 h light and dark cycle) with free access to food and water and acclimatized for two weeks.

### Animal experiments

The sample size (32 SD rats) in this study was determined based on the preset minimum number of rats in each group (*n* = 8), and no a priori sample size calculation was performed before the study.

**Fig. 1:**
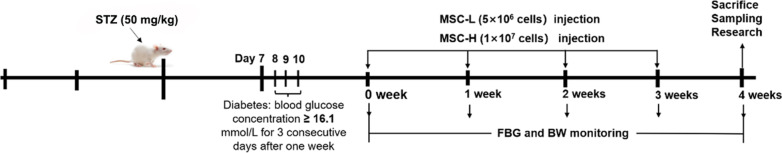
Timetable and flowchart of rat modeling and cell therapy

To evaluate the in vivo efficacy of hucMSCs, 32 rats were randomly (random number table method) divided into four groups (*n* = 8): (i) normal group (Normal), (ii) diabetic model group (Model), (iii) low dose hucMSCs-treated group (MSC-L), and (iv) high dose hucMSCs-treated group (MSC-H). With the animal cage as experimental unit, four units were set and rats in same group were raised in the same cage. Diabetic model was induced by a single intraperitoneal injection of 50 mg/kg streptozocin (STZ) (dissolved in 0.1 mM citrate buffer, pH 4.5) in rats, as described previously [18]. The normal group received equal volume of citrate buffer. The rats with tail blood glucose concentration ≥ 16.1 mM for 3 consecutive days after 1 week were included as successful modeling, otherwise, the rats that did not meet this inclusion criteria were excluded. During the therapy, blood glucose levels of all rats in four experimental unit were monitored once a week, and rats were eliminated if their blood glucose level did not fulfill the aforementioned screening criteria over two weeks. Then, rats in the MSC-L (5 × 10^6^ cells per rat) and MSC-H (1 × 10^7^ cells per rat) groups were injected with hucMSCs intravenously and rats in the normal and model group were injected with equal volume of PBS once a week for 4 weeks in SPF animal room. Body weight (BW) and fasting blood glucose (FBG) were measured weekly. After 4 weeks, urine samples were collected using metabolic cages from the rats for the detection of 24 h urine protein, urine creatinine and urinary albumin/creatinine ratio. After the physical and biochemical measurements, all rats were anesthetized with CO_2_ at a flow rate of 2 ~ 5 l/min and euthanized for the following experiments (Fig. 1).

The rat DN model construction and its following treatments were performed by four experienced operators in a blinded manner. The first operator designed the experimental protocol and grouping. The second operator was responsible for the treatment of all groups of rats. The third operator was responsible for collecting and recording blood glucose, body weight and metabolism of rats in all groups. The fourth investigator anesthetized all animals and collected samples. To reduce possible confounders, treatments were done at the same time and in a random sequence, and all rats in each cage were housed in the same animal room and given ad libitum access to the same food and water.

### Histopathological analysis and immunohistochemical staining

Immediately after euthanasia, kidneys of the rats were removed for weighing. Both sides of kidneys were placed in liquid nitrogen and then preserved in -80℃ for subsequent experiments. And the bilateral kidney were fixed with 4% formaldehyde for 24 h, dehydrated, and embedded in paraffin. Then, 4 μm thick sections were cut in succession and stained using hematoxylin and eosin (HE) and periodic acid Schiff (PAS) for morphology evaluations. For immunohistochemical staining, the paraffin-embedded slices were first dewaxed and antigen retrieval with 0.01 mol/l citrate buffer (pH 6.0, Solarbio, Beijing, China) was performed. After blocking with 8% goat serum for 30 min at room temperature, slices were incubated overnight at 4 °C with p-AMPK and p-mTOR primary antibodies (1:50, Cell Signaling Technology, USA). Subsequently, the sections were treated with horseradish peroxidase-conjugated secondary antibody (PV-9001) for 30 min. After 3,3′-diaminobenzidine (DAB) staining, sections were stained with hematoxylin. For all sections, a microscope was used to obtain the images (Invitrogen EVOS M7000, NY, USA). Image J software (Version 1.49, Bethesda, USA) was used to process the images and quantify the average optical density/area of p-AMPK and p-mTOR. Semiquantitative scoring was performed to assess the degree of renal tissues injury. Vacuolation of tubular epithelial cells and extracellular matrix precipitation were assessed according to the degree of renal tissues injury (score from 0 to 4: 0, normal; 1, minor; 2, mild; 3, moderate and 4, severe), and the renal injury score was calculated by scoring each glomerulus and calculating a weighted average of these scores [22].

### Imaging of fluorescently labeled hucMSCs and tracking

To evaluate tissue distribution and in vivo kinetic of hucMSCs, hucMSCs were stained with 1,1'-dioctadecyl-3,3,3',3'-tetramethylindodicarbocyanine perchlorate (DiD) cell labeling solution (Invitrogen, NY, USA) according to the reagent's instructions and washed twice with PBS. The fluorescently labeled hucMSCs were injected intravenously into rats (5 × 10^6^ cells per rat). At 12 h, 24 h, 48 h, 120 h, and 168 h after injection, rat tissues including kidneys, lungs, liver, spleen, brain, and heart were collected for ex vivo imaging. The intensity of fluorescence was quantified using the IVIS^®^ Spectrum system and Living Image Software (PerkinElmer, Massachusetts, USA).

### Cell culture and conditioned medium preparation

Rat podocytes were purchased from the Chinese Academy of Sciences (Beijing, China) and cultured in Dulbecco’s modified eagle’s medium (DMEM) (Gibco, NY, USA) supplemented with 10% fetal bovine serum (FBS) (Gibco, NY, USA) and 5% penicillin–streptomycin solution in a 37 °C, 5% CO_2_ incubator. HucMSCs were obtained from the Cell Resource Bank and Integrated Cell Preparation Center of Xiaoshan District (Hangzhou, China) and were grown in minimum essential medium-alpha modification (α-MEM) with Glutamax™-1 (Gibco, NY, USA). Conditioned medium (CM) of rat podocytes and of hucMSCs were prepared for in vitro experiments. Briefly, rat podocytes and hucMSCs were seeded at a density of 2 × 10^6^ cells/15 cm dish with complete medium. The medium was refreshed at cell confluency of 80% and the cells were allowed to further grow for 48 h. Then 12 ml of the cell culture medium was collected, centrifuged for 10 min at 1200 rpm. The RP-CM and MSC-CM were filtered through a 0.22 µm cell strainer, and stored at -80℃ for further use.

### In vitro* experiment*

To investigate the effects and action mode of hucMSCs on rat podocytes, both MSC-CM and co-cultured hucMSCs were applied for the in vitro treatment. Rat podocytes were divided into four groups as follows: (i) normal group; (ii) model group; (iii) MSC-CM group, and (iv) MSC group. To establish an in vitro cellular model of high glucose-induced damage, rat podocytes in model and MSC-CM groups were pre-treated with high glucose DMEM (33 mM glucose) for 72 h, while the normal group with normal DMEM (17.5 mM glucose) for 72 h. Subsequently, rat podocytes in the MSC-CM group were treated with MSC-CM for 48 h, while rat podocytes in normal and model groups were treated with RP-CM for 48 h. For co-cultured hucMSCs treatment, rat podocytes were inoculated into 6-well plates and cultured in high glucose DMEM for 72 h. After discarding the supernatant, the upper chamber was placed in the co-culture group and hucMSCs (3 × 10^5^ cells/well) were added to the upper chamber and high glucose DMEM medium was added to the lower chamber, followed by co-culture for another 48 h. Moreover, an autophagy inhibitor chloroquine (CQ) (Selleck, Houston, USA) was applied in the MSC-CM + CQ group to verify the autophagy-related actions of MSC-CM. Rat podocytes in the MSC-CM + CQ group were pre-treated with high glucose DMEM for 72 h, followed by the treatment of MSC-CM containing 70 μM CQ for 48 h. All rat podocytes were washed twice with PBS after intervention for follow-up experiments.

### Cell viability assay

The cell viability of rat podocytes was determined by using Cell Counting Kit-8 (CCK-8) assay. Briefly, cells were seeded into 96-well plates at a density of 2 × 10^3^ cells/well in 200 µl medium, followed by high glucose or MSC-CM intervention as described above. Aliquots of each 20 µl CCK-8 solution (Beyotime, Nanjing, China) were added to each well and incubated at 37 °C for 2 h, until the color turned to orange. The optical density (OD) was measured at 450 nm by a microplate reader (SpectraMax i3x, Shanghai, China). Cell viability was calculated according to the following formula: [(OD value of each group/ average OD value of the normal group) × 100%]. Each experiment was conducted in triplicate.

### Wounding healing assay

To perform wounding healing assay, rat podocytes in the logarithmic growth phase were inoculated into six-well plates at a density of 4 × 10^4^ cell/well. The cell monolayer was then scraped with sterile 100 µl pipette tips to form a cell-free rectangular zone, followed by high glucose or MSC-CM treatment as described above and the mannitol group was set up as an osmotic pressure control (17.5 mM glucose plus 15.5 mM mannitol). Then, cells were washed twice with PBS and replaced with fresh serum-free medium. The cells were observed and imaged at two different time points (0 h and 24 h) by using an inverted microscope (Olympus IX73, Japan). The scratch area was measured by using ImageJ software (Version 1.49, Bethesda, USA). Each experiment was conducted in triplicate.

### Cell senescence assay

Cell senescence of rat podocytes was observed by using senescence-associated β-galactosidase (SA-β-gal) staining. Briefly, cells were seeded in six-well plates (4 × 10^4^ cell/well), followed by high glucose or MSC-CM treatment as described above. Cells were then fixed with 2% formaldehyde and stained using SA-β-gal staining kit (Beyotime, Nanjing, China), according to the manufacturer’s instructions. The percentage of SA-β-gal-positive cells was calculated as (the number of SA-β-gal-positive cells / the total number of cells) × 100%. Each experiment was conducted in triplicate.

### Real-time PCR (qPCR) analysis

Total RNA from rat podocytes was extracted by using RNAiso Plus reagent (Takara Bio, Beijing, China), quantified by a NanoDrop™ 2000 spectrometer (Thermo Fisher Scientific, MA,USA), and reverse transcribed to cDNA by using a PrimeScript™ RT reagent kit (Takara Bio, Beijing, China). The cDNA was amplified by using the TB Green PCR kit (Takara Bio, Beijing, China). qPCR was performed on LightCycler^®^ 480 system (Roche, Shanghai, China) in a 20 µl qPCR reaction system with the following procedure: pre-incubation at 95 °C for 5 min, followed by 40 cycles of denaturation at 95 °C for 10 s, annealing and extension at 60 °C for 30 s.* GAPDH* was used as a reference gene and the primer sequences for all genes are shown in Table 1. The relative mRNA expression was measured by the 2^−∆∆Cq^ method. Each experiment was conducted in triplicate.

**Table 1:** Primer sequences used for qPCR analysis

Gene	Forward primer	Reverse primer
*GAPDH*	5'-GACATGCCGCCTGGAGAAAC-3'	5'-AGCCCAGGATGCCCTTTAGT-3'
*p16*	5'-TCCTTGGCTTCACTTCTGGCAAC-3'	5'-TCCTTGGCTTCACTTCTGGCAAC-3'
*p21*	5'-GAAAACGGAGGCAGACCAG-3'	5'-TTCAGGGCTTTCTCTTGCAG-3'
*p53*	5'-GTCTACGTCCCGCCATAAAA-3'	5'-AGGCAGTGAAGGGACTAGCA-3'
*pRb*	5'-CTTGCGGATTCCTGGAGGTAACATC-3'	5'-TCCCAAATGATTCACCGATTGAGACC-3'
*p62*	5'-GGTGTCTGTGAGAGGACGAGGAG-3'	5'-TCTGGTGATGGAGCCTCTTACTGG-3'
*Beclin-1*	5'-TCAAGATCCTGGACCGAGTGACC-3'	5'-CTCCTCTCCTGAGTTAGCCTCTTCC-3'
*LC3B*	5'-GAGCGAGTTGGTCAAGATCATCCG-3'	5'-GATGTCAGCGATGGGTGTGGATAC-3'
*ULK1*	5'-TACACAGCAAGGGCATCATTCACC-3'	5'-CGGGCAAATCCAAAGTCAGCAATC-3'

### Western blot (WB) analysis

The protein expressions of rat podocytes and kidney tissues were determined by using western blot (WB) analysis. Total protein of rat podocytes from each group was extracted with RIPA lysis buffer (Beyotime, Nanjing, China). To ensure the reproducibility of the in vivo WB assays, kidney samples were obtained from three separate rats in the normal, model, MSC-L, and MSC-H groups, respectively. For the protein isolation of the kidney, part of the rat kidney tissue was cut with sterilized scissors and placed in lysate with multi-tissue homogenizer (Tissuelyser-24, Shanghai, China) at 65 Hz for 90 s, followed by lysis at 4℃ for 30 min. The lysate was centrifuged at 4℃ for 15 min, and the supernatant was transferred to the precooled centrifuge tube. The protein concentration was determined by using Bicinchoninic Acid (BCA) Kit (Beyotime, Nanjing, China). 30 μg total protein lysate was loaded onto 10% or 15% sodium dodecyl sulfate polyacrylamide gel electrophoresis (SDS-PAGE) gels for separation and transferred to polyvinylidene difluoride (PVDF) membranes (Merck Millipore, MA, USA). The membranes were blocked with 5% skimmed milk for 2 h, followed by incubation overnight at 4℃ with primary antibodies. The primary antibodies used in this experiment are shown in Table 2. The membranes were then probed with the corresponding horse radish peroxidase (HRP)-conjugated goat anti-rabbit IgG (1:2000, cat. no. 14708; Cell Signaling Technology) secondary antibody for 90 min at room temperature. Bands were visualized with ECL reagent (Biological Industries) and blots were displayed on X-ray film (Beyotime, Nanjing, China) and detected by Gel Doc XR + Imaging Systems (Bio-Rad, California, USA). The density of each strip was analyzed by using ImageJ software (version 1.49, Bethesda, USA). Each experiment was conducted in triplicate.

**Table 2:** Antibodies used for WB analysis

Antibodies	Host	Dilution	Company and location
p16	Rabbit	1:1000 for WB	Abcam, USA
p53	Rabbit	1:1000 for WB	Cell Signaling Technology, USA
p62	Rabbit	1:1000 for WB	Cell Signaling Technology, USA
Beclin-1	Rabbit	1:1000 for WB	Cell Signaling Technology, USA
LC3B	Rabbit	1:1000 for WB	Cell Signaling Technology, USA
ULK1	Rabbit	1:1000 for WB	Abcam, USA
AMPK	Rabbit	1:1000 for WB	Cell Signaling Technology, USA
p-AMPK	Rabbit	1:1000 for WB	Cell Signaling Technology, USA
mTOR	Rabbit	1:1000 for WB	Cell Signaling Technology, USA
p-mTOR	Rabbit	1:1000 for WB	Cell Signaling Technology, USA
GAPDH	Rabbit	1:1000 for WB	Cell Signaling Technology, USA

### Statistical analysis

All data were analyzed by using SPSS 22.0 software (SPSS, Chicago, USA). Firstly, the analysis data were tested for normality. If the data conformed to normality, the Student′s *t* test was performed when analyzed the statistical significance of two groups and the one-way analysis of variance (ANOVA) based on the least significant difference (LSD) method was used for the multiple comparisons analysis. If the data did not conform to normality, nonparametric statistics were applied. Data were expressed as mean ± standard deviation (SD). Differences were considered statistically significant when *P* < 0.05 and < 0.01.

## Results

### Characterization of hucMSCs

**Fig. 2:**
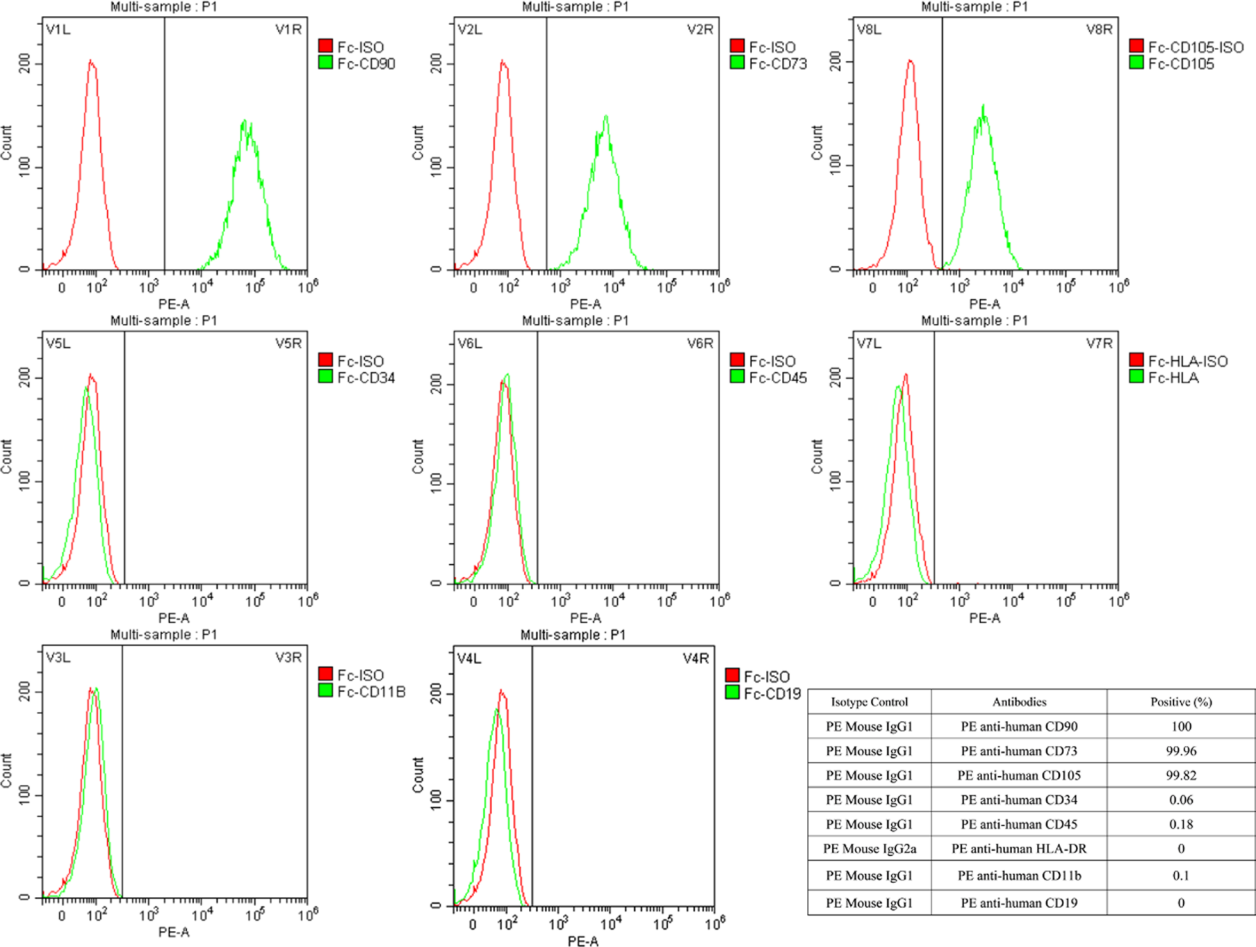
The immunophenotype of hucMSCs determined by flow cytometry

Flow cytometry results showed that the cultured hucMSCs were positive for CD73 (99.96%), CD90 (100%), and CD105 (99.82%) markers and were negative for CD34 (0.06%), CD45 (0.18%), HLA-DR (0%), CD11b (0.1%), and CD19 (0%) markers, which was consistent with the typical phenotype of MSCs (Fig. 2).

### HucMSCs improved renal dysfunction in diabetic rats

**Fig. 3:**
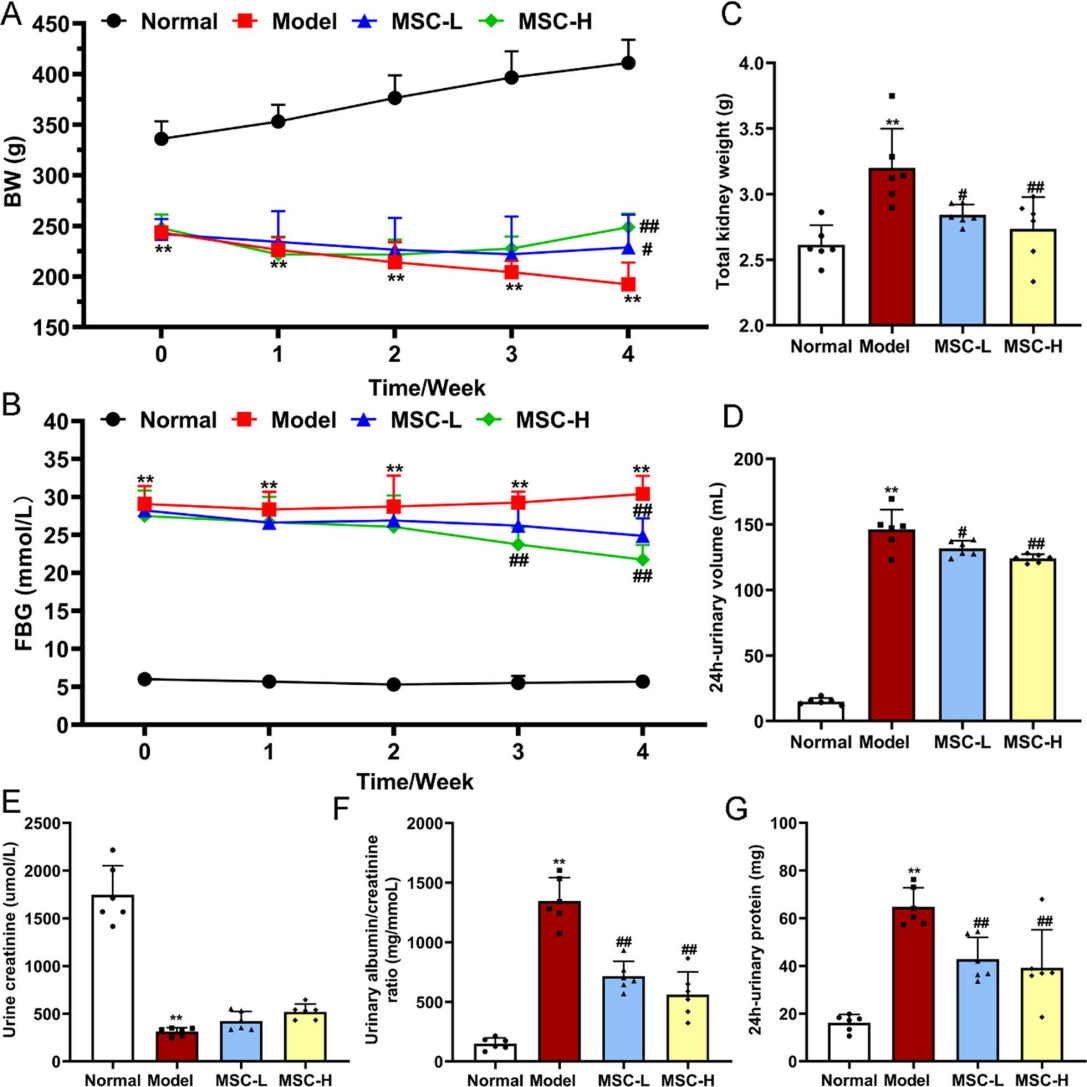
Physical and biochemical analysis of rats. Body weight (BW) (**A**), fasting blood glucose (FBG) (**B**), total kidney weight (**C**), 24 h urine volume (**D**), 24 h urinary protein (**E**), urine creatinine (**F**), and urinary albumin/creatinine ratio (**G**) in the normal (*n* = 6), model (*n* = 6), MSC-L (*n* = 6), and MSC-H (*n* = 6) groups. Data were shown as mean ± SD, *n* = 6, unpaired, student’s *t* test and one-way ANOVA. *P < 0.05 and **P < 0.01 versus the normal group, ^**#**^*P* < 0.05 and ^**##**^*P* < 0.01 versus the model group

To investigate the therapeutic efficacy of hucMSCs on DN, a diabetic rat model was established by STZ injection. After modeling, a sustained decrease in body weight and a significant increase in FBG were observed in the model group of rats (each *P* < 0.01 vs. normal) (Fig. 3A, B). The changes in the model group were accompanied by a significant increase in bilateral kidney weight, 24 h urine volume, 24 h urine protein and urine albumin/creatinine ratio, as well as a significant decrease in urine creatinine level of rats (each *P* < 0.01 vs. normal) (Fig. 3C, D, E, F, and G) and (Additional file 1: Fig. S1). By contrast, 4 weeks after injections of hucMSCs, rats in the MSC-L and MSC-H groups had significantly higher body weight and significantly lower FBG compared to the model group (each *P* < 0.05 or *P* < 0.01 vs. model) (Fig. 3A, B). In addition, the renal function parameters (bilateral kidney weight, 24 h urine volume, 24 h urine protein and urine albumin/creatinine ratio) were also significantly restored in the MSC-L and MSC-H groups after the hucMSCs treatment (each *P* < 0.05 or *P* < 0.01 vs. model) (Fig. 3C, D, F, and G) and (Additional file 1: Fig. S1). There was a modest but insignificant increase in urinary creatinine levels in the MSC-L and MSC-H group (Fig. 3E). Taken together, the above results indicated that renal function was impaired in STZ-induced diabetic rats, while hucMSCs were effective in restoring not only abnormal blood glucose but also renal dysfunction in diabetic rats.

### HucMSCs alleviated renal histological damage in diabetic rats

**Fig. 4:**
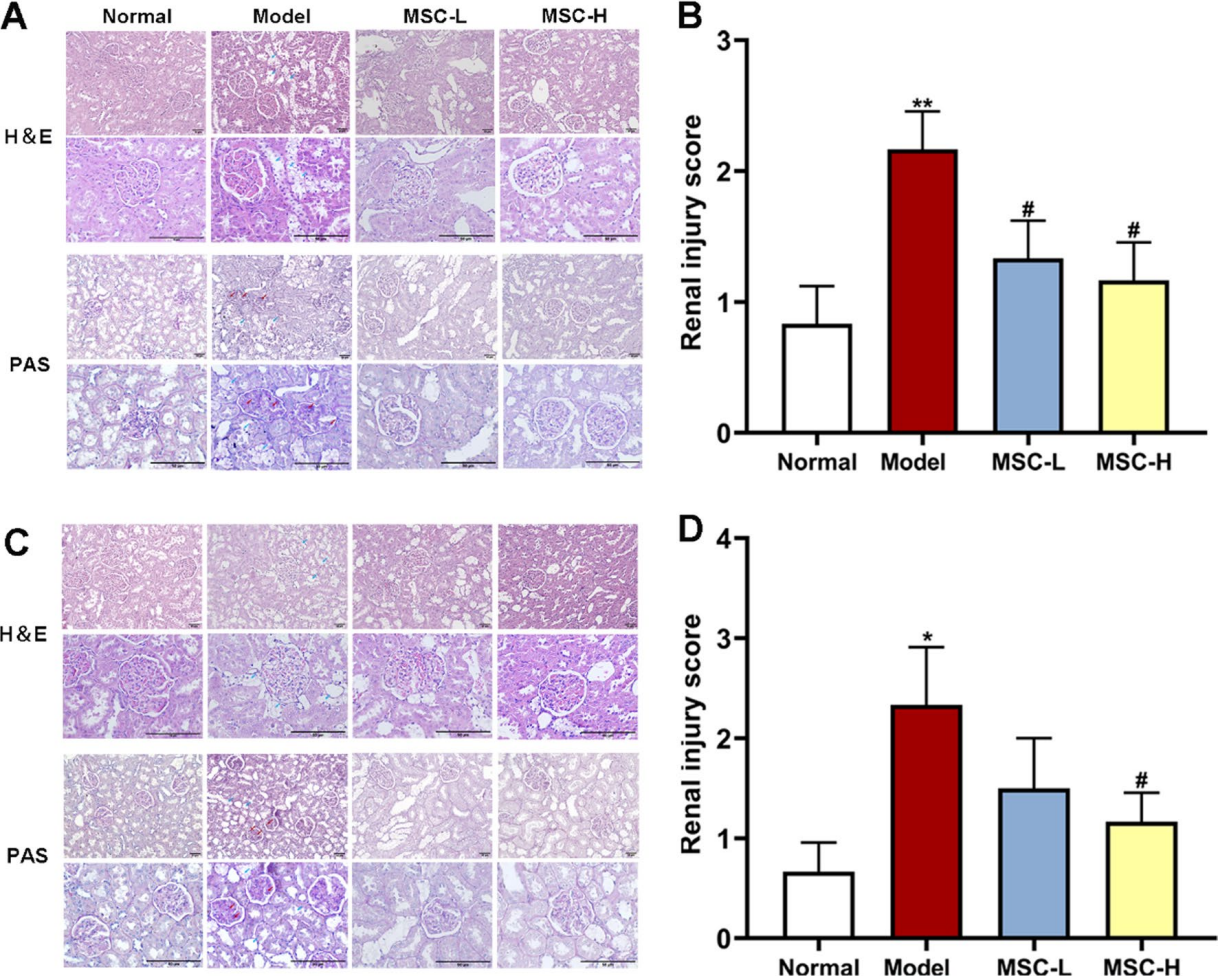
Effects of hucMSCs on kidney morphology in diabetic rats. **A** and **B** Representative micrographs and renal injury score of PAS-stained and HE-stained of left renal tissues (200× and 400×) (scale bar = 50 μm). **C** and **D** Representative micrographs and renal injury score of PAS-stained and HE-stained of right renal tissues (200× and 400×) (scale bar = 50 μm). Red arrows indicate vacuolation of tubular epithelial cells, and blue arrows indicate glomerular vacuolar degeneration. Data were shown as mean ± SD, *n* = 3, unpaired, student's *t* test. *P < 0.01 versus the normal group, ^**#**^*P* < 0.05 and ^**##**^*P* < 0.01 versus the model group

Histopathological observation was performed to evaluate the repairing effect of hucMSCs on the diabetic renal damage. The HE and PAS staining showed significant vacuolation of renal tubular epithelial cells, and slightly enhanced glomerular basement membrane and extracellular matrix deposition in both kidneys of the model group (Fig. 4A, C). However, the above pathological abnormalities were alleviated in the MSC-L and MSC-H groups (Fig. 4A, C). The renal injury score kidney was increased in the model group than that in the normal group (each *P* < 0.01 vs. normal) (Fig. 4B, D). By contrast, intravenous injections of hucMSCs apparently alleviated the above pathological abnormalities, with decreased renal injury score in both the MSC-L and MSC-H groups (each *P* < 0.05 vs. model) (Fig. 4B, D). Consistent with the physical and biochemical changes described above, these histopathological results suggested that hucMSCs alleviated renal damage in diabetic rats.

### Effects of hucMSCs on renal protein expressions in diabetic rats

**Fig. 5:**
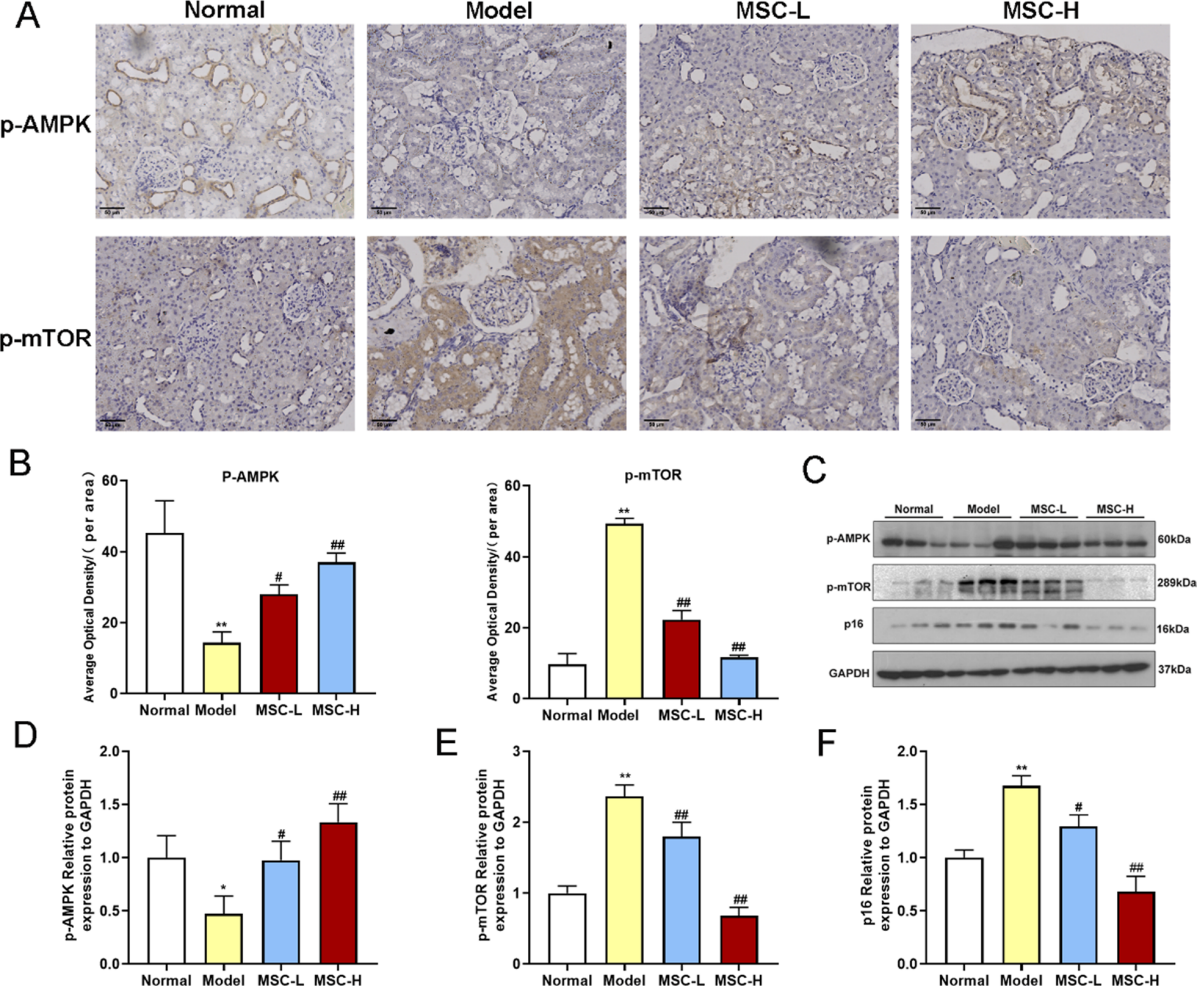
Effects of hucMSCs on renal protein expressions in diabetic rats. **A** and **B** The representative images (200×) (scale bar = 50 μm) and the average optical density values of immunohistochemical staining of p-AMPK and p-mTOR. **C** Representative images of p-AMPK, p-mTOR, p16, and GADPH in renal tissues in WB analysis. Full-length blots of renal tissue were presented (Additional file 2: Figs. S26–S29 and Additional file 3: Fig. S34), respectively. **D**, **E** and **F** Relative protein expressions of p-AMPK, p-mTOR, and p16 to GADPH in WB analysis. Data were shown as mean ± SD, *n* = 3, unpaired, student's *t* test. *P < 0.05 and **P < 0.01 versus the normal group, ^**#**^*P* < 0.05 and ^**##**^*P* < 0.01 versus the model group

As shown in Fig. 5, IHC staining showed that the expression of p-AMPK was significantly decreased and the expression of p-mTOR was significantly increased in the renal tissues of model group (each *P* < 0.05 or *P* < 0.01 vs. normal), while those were markedly restored in the MSC-L and MSC-H groups (each *P* < 0.05 or *P* < 0.01 vs. model) (Fig. 5A, B). Furthermore, WB analysis demonstrated that the above results were reconfirmed due to the similar results of p-AMPK and p-mTOR protein expressions (Fig. 5C, D and E). Moreover, the expression of p16 was significantly increased in the model group (each *P* < 0.01 vs. normal), which was markedly improved in the MSC-L and MSC-H groups (each *P* < 0.05 or *P* < 0.01 vs. model) (Fig. 5C, F).

### In vivo* kinetic observation of hucMSCs by fluorescence imaging*

**Fig. 6:**
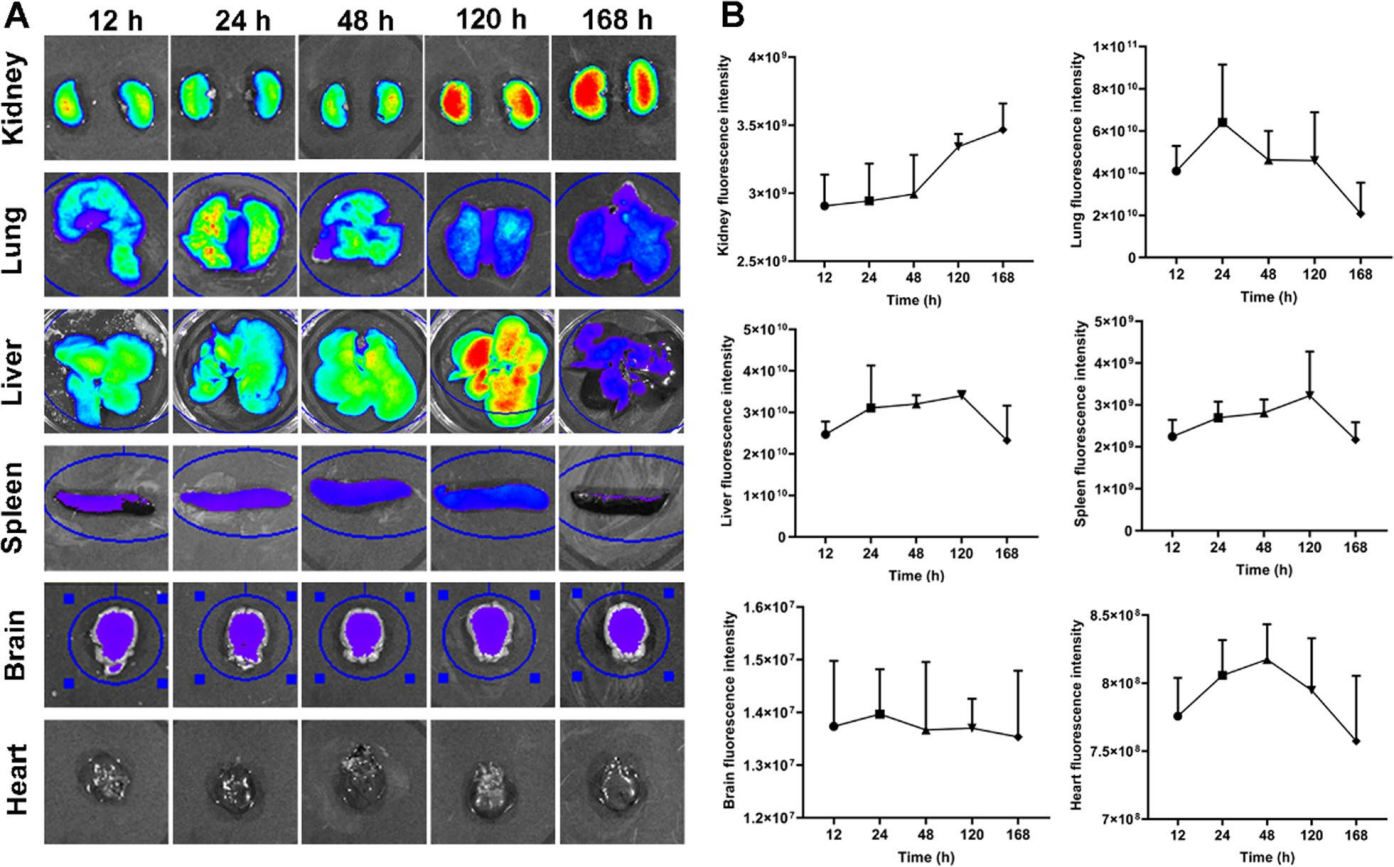
The distribution of hucMSCs in various organs in rats within 168 h/7 d. **A** Representative ex vivo bioluminescence images. **B** Quantifications of each tissue at different time points. Data were shown as mean ± SD, *n* = 3

Ex vivo imaging of the dissected organs revealed that the DiD-labeled hucMSCs were mainly distributed in lungs, liver, and kidneys of rats (Fig. 6A). The fluorescence quantitation revealed that the accumulation of hucMSCs was increased in each organ at 24 h after injection, and the accumulated hucMSCs were decreased in lungs, liver, spleen, brain, and heart at 48 h, 168 h, 168 h, 48 h, and 120 h, respectively (Fig. 6B). Interestingly, the distribution of hucMSCs in kidneys remained increasing even at 168 h after injection, indicating that hucMSCs tended to accumulate at the kidney.

### HucMSCs improved abnormal viability, scratched damage, and senescence of diabetic podocytes in paracrine mode

**Fig. 7:**
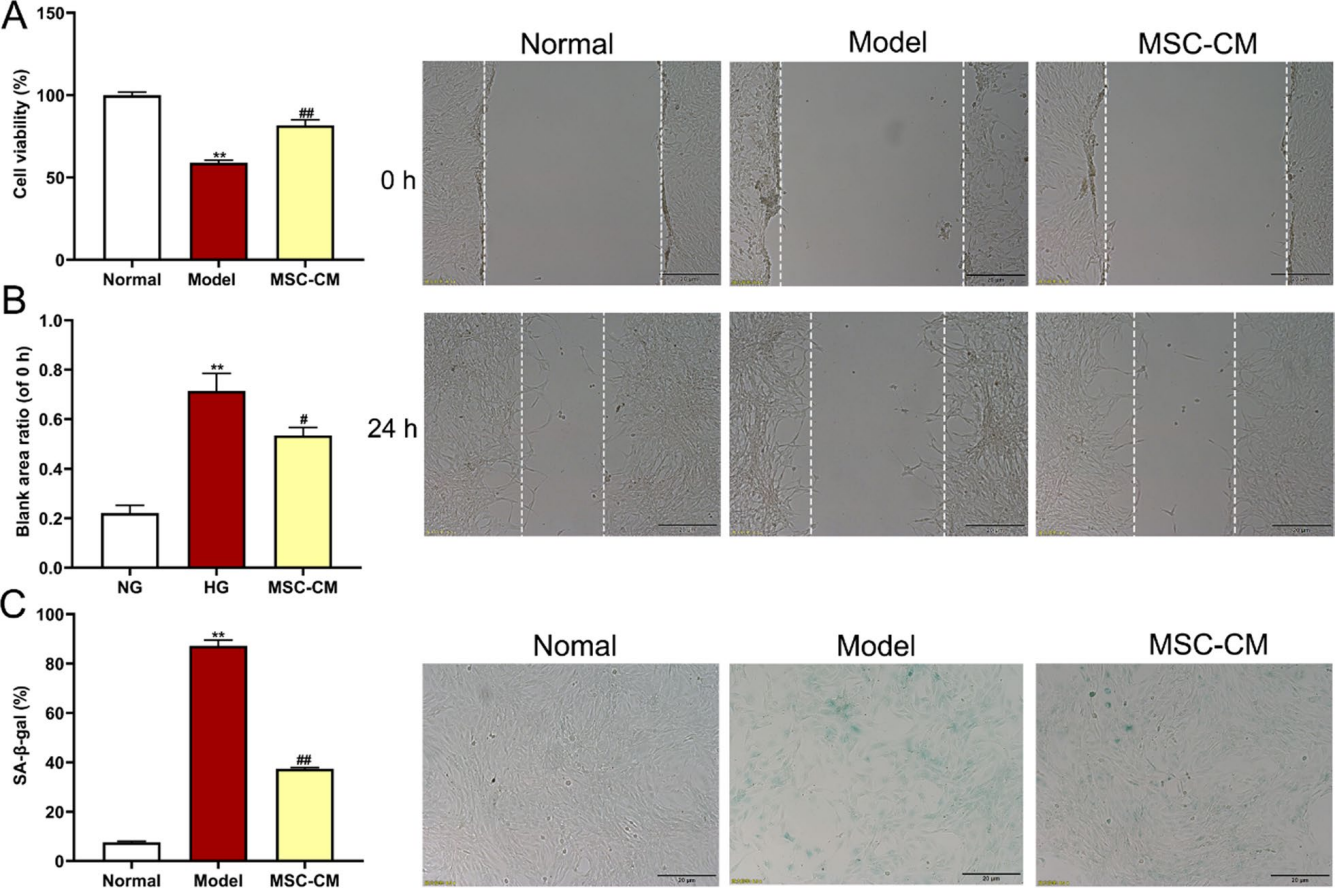
Effects of MSC-CM on cell viability, wound healing, and cell senescence of rat podocytes in the normal, model, and MSC-CM groups. **A** CCK-8 assay for cell viability. **B** Wound healing assay and representative pictures in the scratch test (scale bar = 20 μm). **C** Representative images of senescence-associated β-galactosidase (SA-β-gal) staining for renal podocytes cells and the percentage of SA-β-gal-positive cells in each group (scale bar = 20 μm). Data were shown as mean ± SD, *n* = 3, unpaired, student's *t* test. **P < 0.01 versus the normal group, ^**#**^*P* < 0.05 and ^**##**^*P* < 0.01 versus the model group

**Fig. 8:**
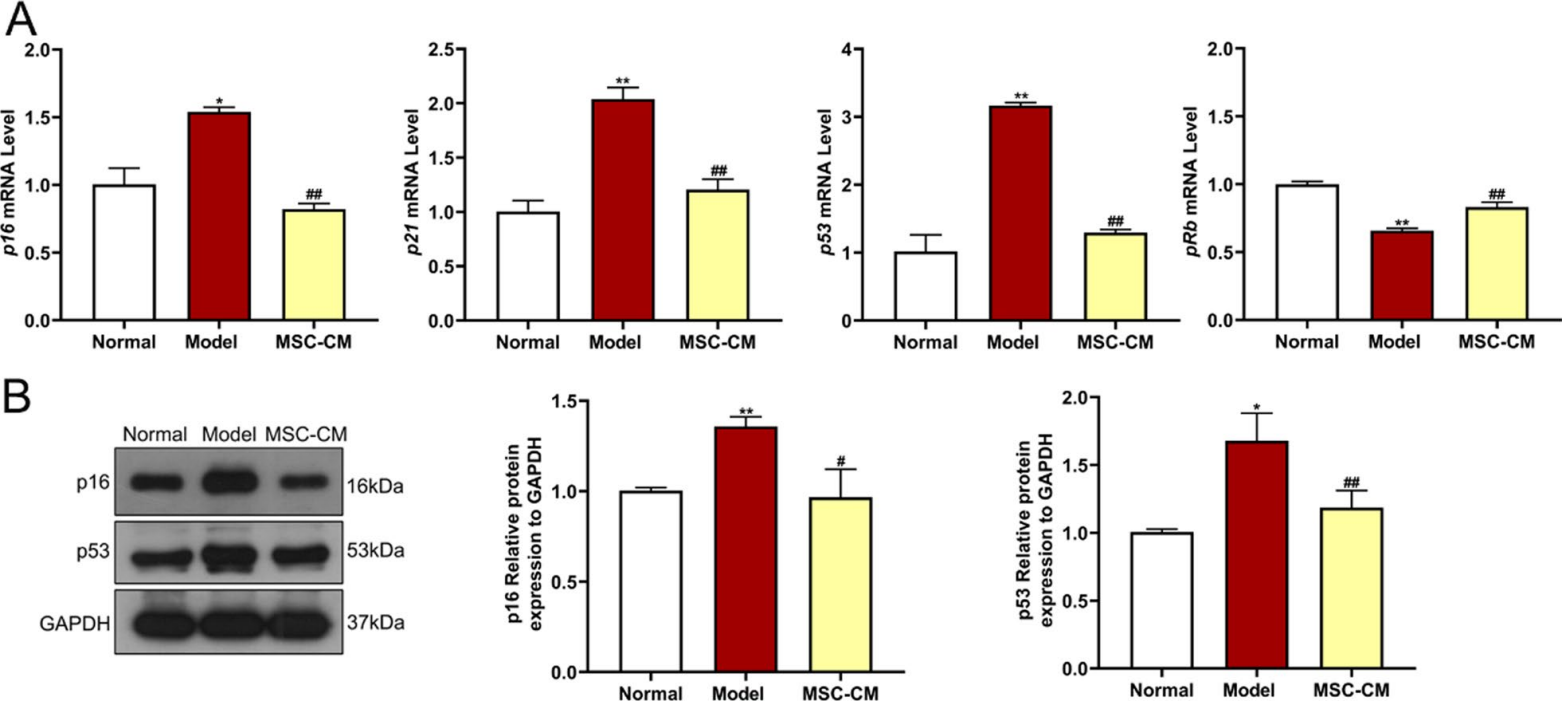
Molecular regulations of MSC-CM on high glucose-induced senescence of rat podocytes. **A** qPCR was performed to detect the expressions of senescence-related genes, including senescence markers *p16*, *p21*, *p53*, and *pRb*. **B** WB was performed to measure the expression of senescence-related proteins, including p16 and p53. Full-length blots of rat podocytes were presented (Additional file 2: Figs. S5–S7), respectively. Data were shown as mean ± SD, *n* = 3, unpaired, student’s *t* test. *P < 0.05 and **P < 0.01 versus the normal group, ^**#**^*P* < 0.05 and ^**##**^*P* < 0.01 versus the model group

To initially investigate how hucMSCs repaired the damaged renal tissues, rat podocytes were applied to evaluate the in vitro effects of MSC-CM on cell viability, wound healing, senescence, and gene expressions of podocytes under high glucose stress. This study compared the effects of glucose on renal podocytes between different glucose concentrations. CCK8 and WB results showed no significant difference in cell viability and senescence protein expressions between 5 mM and 17.5 mM glucose, while the cell viability and senescence protein expressions were significantly reduced at 33 mM and 42.5 mM glucose were compared to 17.5 mM glucose (each *P* < 0.01 vs. 17.5 mM). Therefore, we used 17.5 mM glucose in the normal group and 33 mM glucose in the high glucose group as a model group (Additional file 1: Figure S2, Additional file 2: Figs. S23–S25). High glucose (33 mM) not only significantly decreased the cell viability of podocytes, but also increased the percentage of wound area ratio (24 h/0 h) and β-gal-stained senescent podocytes in the model group (each *P* < 0.01 vs. normal) (Fig. 7A, B and C). By contrast, the MSC-CM treatment significantly improved the above abnormalities (each *P* < 0.05 or *P* < 0.01 vs. model) (Fig. 7A, B and C). In addition, we used the mannitol group as the osmotic pressure control, demonstrated the inhibition of podocytes motility following high glucose intervention (Additional file 1: Figure S3). The qPCR results showed that high glucose significantly increased the expressions of *p16*, *p21*, *p53*, and decreased the expression of *pRb* in the model group (each *P* < 0.05 or *P* < 0.01 vs. normal) (Fig. 8A). By contrast, the MSC-CM treatment significantly restored the above abnormalities of gene expressions in the model group (each *P* < 0.05 or *P* < 0.01 vs. model) (Fig. 8A). Consistent with the qPCR results, the WB results indicated that high glucose significantly up-regulated the protein expressions of p16 and p53 (each *P* < 0.05 or *P* < 0.01 vs. normal) and MSC-CM significantly restored the abnormal expressions (each *P* < 0.05 or *P* < 0.01 vs. model) (Fig. 8B). The above results indicated that hucMSCs restored the high glucose-induced abnormalities of cell viability, scratched damage, senescence state, and senescence-related gene expressions of rat podocytes in a paracrine mode.

### HucMSCs improved abnormal senescence of diabetic podocytes in co-culture mode

**Fig. 9:**
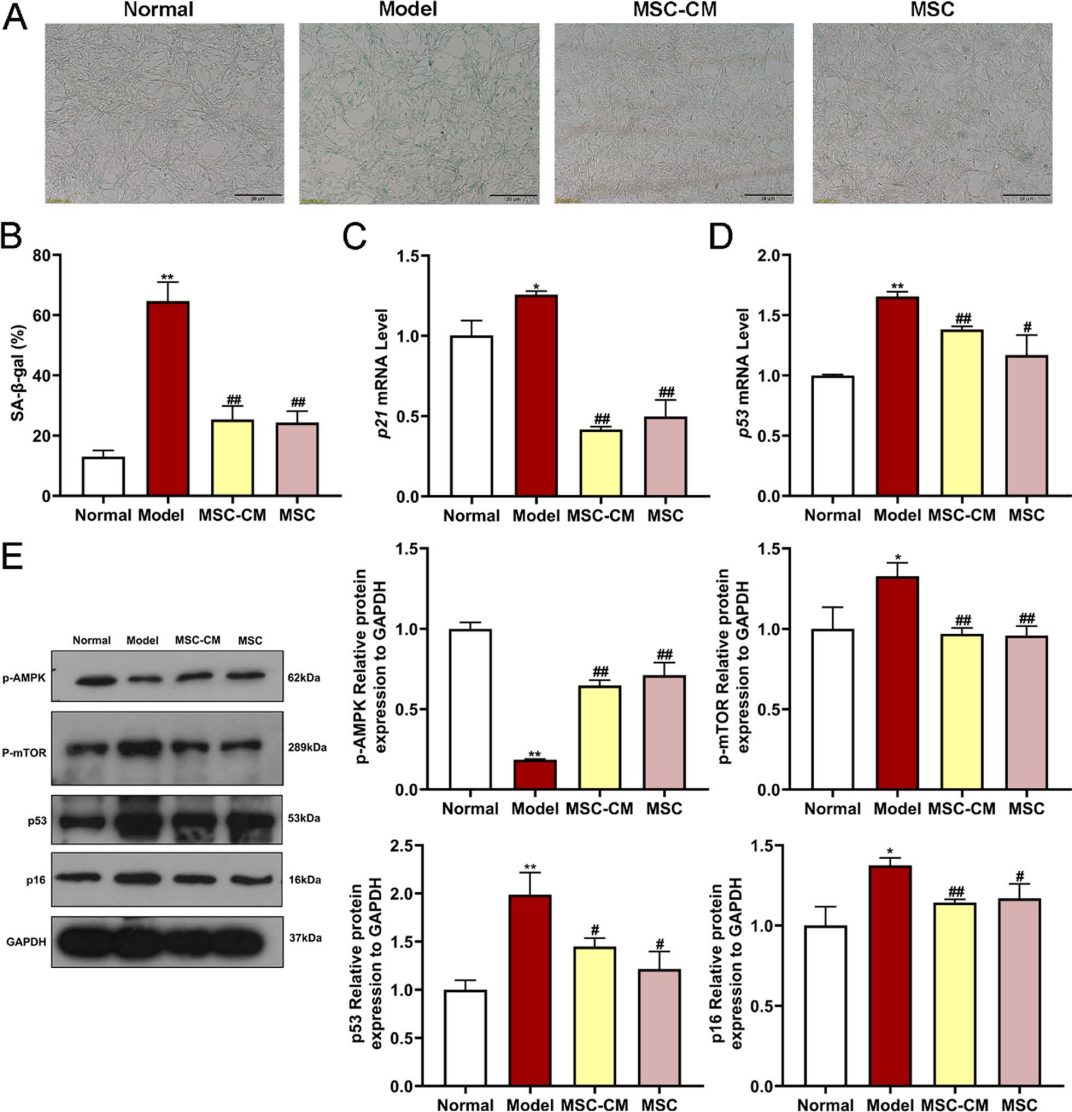
Effects of hucMSCs on cell senescence, mRNA levels, and protein expressions of podocytes under co-culture conditions. **A** and **B** Representative images of SA-β-gal staining on rat podocytes and the percentage of SA-β-gal-positive cells in each group (scale bar = 20 μm). **C** and **D** qPCR was performed to detect the expressions of senescence-related genes, including senescence markers *p21*and *p53*. (E) WB was performed to measure the expressions of autophagic pathway and senescence-related proteins, including p-AMPK, p-mTOR, p16, and p53. Full-length blots of rat podocytes were presented (Additional file 2: Figs. S8–S11, Additional file 3: Figs. S30–S33), respectively. Data were shown as mean ± SD, *n* = 3, unpaired, student's *t* test. *P < 0.05 and **P < 0.01 versus the normal group, ^#^*P* < 0.05 and ^##^*P* < 0.01 versus the model group

To confirm the paracrine action of hucMSCs on rat podocytes, a co-culture mode was applied in cell senescence staining, qPCR and WB assays, and a comparison was made with the conditioned medium. The number of β-gal-stained senescent podocytes in the model group was significantly higher than that in the normal group (*P* < 0.01 vs. normal), while that in the MSC-CM group and the MSC group were significantly decreased (*P* < 0.01 vs. model) (Fig. 9A and B). The qPCR results showed that high glucose significantly increased the mRNA expressions of *p21* and *p53* (each *P* < 0.05 or *P* < 0.01 vs. normal), while those expressions in the MSC-CM group and the MSC group were significantly reversed (each *P* < 0.05 or *P* < 0.01 vs. model) (Fig. 9C and D). The WB results indicated that high glucose significantly up-regulated the protein expressions of p-AMPK, p-mTOR, p16, and p53 (each *P* < 0.05 or *P* < 0.01 vs. normal), while those expressions in the MSC-CM group and the MSC group were significantly reversed (each *P* < 0.05 or *P* < 0.01 vs. model) (Fig. 9E). The above data demonstrated that the co-cultured hucMSCs exerted similar effects as the conditioned medium of hucMSCs.

### AMPK/mTOR signaling in autophagy mediated the paracrine mechanism of hucMSCs on podocytes

**Fig. 10:**
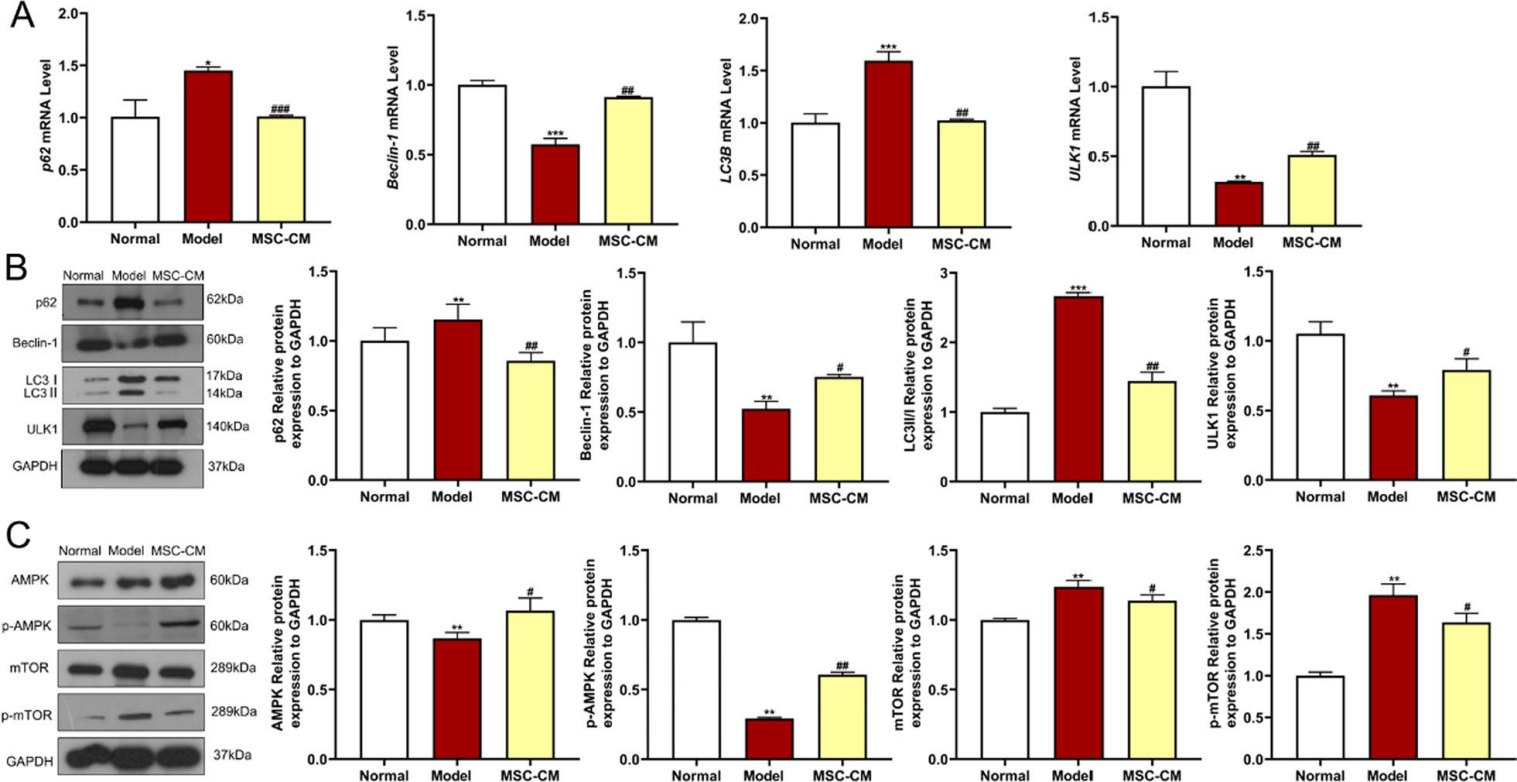
Effects of MSC-CM on autophagic flow of rat podocytes induced by high glucose. **A** qPCR was performed to detect the expression of autophagy-related genes, including *Beclin-1*, *p62*, *LC3B*, and *ULK1*. **B** WB was performed to measure expression of autophagy-related proteins including Beclin-1, p62, LC3, and ULK1. **C** Expressions of autophagic pathway proteins, including AMPK, p-AMPK, mTOR and p-mTOR. Full-length blots of rat podocytes were presented (Additional file 2: Figs. S12–S16), respectively. Data were shown as mean ± SD, *n* = 3, unpaired, student’s *t* test. *P < 0.05 and **P < 0.01 versus the normal group, ^**#**^*P* < 0.05 and ^**##**^*P* < 0.01 versus the model group

**Fig. 11:**
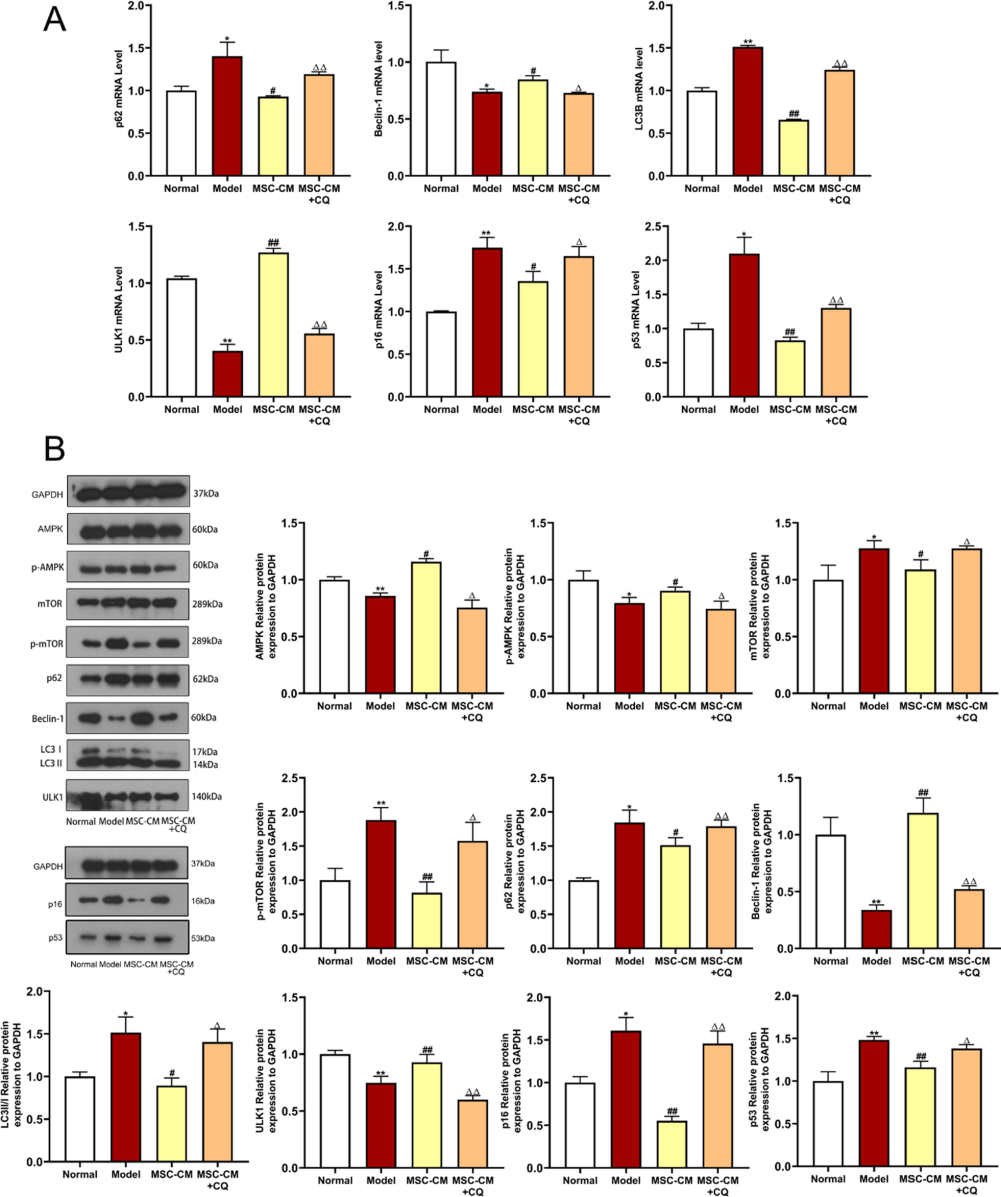
Effects of MSC-CM on aging of rat podocytes in vitro by promoting autophagy. **A** qPCR detection of the mRNA expressions of autophagy (*Beclin-1, p62, LC3B* and *ULK1*) and senescence (*p16* and *p53*)-related genes in rat podocytes after CQ intervention. **B** WB detection of the expressions of AMPK/mTOR pathway autophagy (AMPK, p-AMPK, mTOR, p-mTOR, Beclin-1, p62, LC3 and ULK1) and senescence (p16 and p53)-related proteins in rat podocytes after CQ intervention. Full-length blots of rat podocytes were presented (Additional file 2: Figs. S17–S22), respectively. Data were shown as mean ± SD, *n* = 3, unpaired, student's *t* test. *P < 0.05 and **P < 0.01 versus the normal group, ^**#**^*P* < 0.05 and ^**##**^*P* < 0.01 versus the model group, ^△^*P* < 0.05 and ^△△^*P* < 0.01 versus the MSC-CM group

To explore the paracrine mechanism of hucMSCs on DN, we further investigated the regulative actions of MSC-CM at molecular levels. The qPCR and WB results showed that high glucose significantly decreased the mRNA and protein expressions of *Beclin-1* and *ULK1*, and increased the expressions of *p62* and *LC3B* in the model group (each *P* < 0.05 or *P* < 0.01 vs. normal) (Fig. 10A and B). The high glucose dysregulated expressions were significantly restored by MSC-CM treatment (each *P* < 0.05 or *P* < 0.01 vs. model) (Fig. 10A and B). Moreover, high glucose also significantly decreased the protein expressions of AMPK and p-AMPK, and increased the expressions of mTOR and p-mTOR in the model group (each* P* < 0.05 or *P* < 0.01 vs. normal), while MSC-CM treatment significantly restored these abnormal expressions (each* P* < 0.05 or *P* < 0.01 vs. model) (Fig. 10C). For verification, CQ was used to counteract the actions of MSC-CM. The qPCR and WB results showed that combined treatment of MSC-CM and CQ significantly reversed the regulative actions of MSC-CM on the autophagy and senescence genes (each *P* < 0.05 or *P* < 0.01 vs. MSC-CM) (Fig. 11A and B). CQ not only counteracted the pro-autophagy regulation of MSC-CM on AMPK, p-AMPK, mTOR, p-mTOR, p62, Beclin-1, LC3II/I, and ULK1, but also the anti-senescent regulation of MSC-CM on p16 and p53 (each* P* < 0.05 or *P* < 0.01 vs. MSC-CM) (Fig. 11B). The above results indicated that the high glucose-induced senescence of rat podocytes was due to defective autophagy, and hucMSCs in paracrine mode (MSC-CM) attenuated the senescence by activating autophagy through AMPK/mTOR pathway.

## Discussion

Recently, a growing number of animal studies and clinical trials have indicated the significant therapeutic potential of hucMSCs for diabetes [26]. A recent phase II clinical trial confirmed the efficacy and safety of hucMSCs for adults with type 2 diabetes, as demonstrated by significantly reduced glycated hemoglobin levels at 48 weeks after hucMSCs treatment (1.31%, *P* < 0.01 vs. before treatment) [27]. In the present study, we observed about 30% reduction in blood glucose in diabetic rats with hucMSCs injection (*P* < 0.01 vs. model) (Fig. 3), which validated the previous findings of the anti-diabetes potential of hucMSCs. Even so, the hypoglycemic efficacy of MSCs was not satisfactory and thereby MSCs could not substitute the current antidiabetics [28]. Other studies have reported that MSCs could benefit renal regeneration through anti-inflammatory and anti-fibrosis actions, suggesting that MSCs have potential in treating DN [23, 29, 30]. A clinical trial has tried a single intravenous injection of ADSCs to treat 30 patients with DN for three months, and the outcomes showed recovery of renal function with little adverse event [31]. However, the comprehensive evaluation of the efficacy, tolerability, and safety of MSCs in the treatment of DN is still lacking [32]. Therefore, this study conducted evaluation of the concrete efficacy and mechanism of hucMSCs in the treatment of DN by applying in vivo (STZ-induced diabetic rats) and in vitro (high glucose-induced rat podocytes) models. The in vivo data showed that hucMSCs exerted ameliorative effects on elevated urinary protein and creatinine levels and reduced renal damages in DN rats (Figs. 3 and 4). The in vitro data showed that hucMSCs not only improved the cell viability and damage repair ability of podocytes, but also ameliorated the senescence state of podocytes under high glucose conditions (Fig. 7). Moreover, we found that AMPK/mTOR pathway-dependent autophagy activation mediated the protective and anti-senescent effects of hucMSCs on podocytes (Figs. 8, 9 and 10). By using the conditioned medium, the protective and anti-senescence effects on podocytes were determined as the paracrine action of hucMSCs. The innovative points of this study were as follows: (1) determination of therapeutic efficacy of hucMSCs on blood glucose and DN in diabetic rats; (2) revealing of hucMSCs’ paracrine actions of protection and anti-senescence on podocytes; and (3) discovery of AMPK/mTOR signaling pathway-dependent pro-autophagy mechanism of hucMSCs on DN. Previous studies have found that the paracrine action of MSCs was mainly attributed to the secretion of extracellular vesicles (EVs) and soluble secretions [33]. Since the diameter of EVs is 50–150 nm [33], the filter used in this study might not remove EVs from the conditioned medium, suggesting that both EVs and soluble factors contributed to the paracrine action of hucMSCs.

Podocytes are highly differentiated, and unique glomerular epithelial cells that are critical components of the glomerular filtration barrier [34]. Hyperglycemia could cause oxidative stress in diabetic patients, which could promote senescence of podocytes and accelerate kidney damage [8, 9]. The senescence or related loss of podocytes could cause vicious feedback that eventually leads to DN [35]. Many studies have shown that the number of senescent podocytes was significantly increased in diabetes animal models and DN patients [36, 37]. Likewise, this study found that high glucose-induced podocytes senescence and hucMSCs restored the senescent state to normal. In addition, we found that AMPK/mTOR pathway-associated autophagy mediated the senescence of DN. The immunohistochemistry and WB analysis of rat kidney tissue samples results showed that hucMSCs significantly improved the down-regulation of p-AMPK and up-regulation of p-mTOR, and significantly inhibited the expression of senescence-related protein p16. These results indicated that hucMSCs could improve the aging of renal tissues in diabetes rats through AMPK/mTOR pathway, which partly verified the in vitro mechanisms of hucMSCs in DN treatment. The mTOR signaling pathway is the main negative regulator of autophagy, and podocytes exhibited high levels of basal autophagy under normal physiological conditions [38, 39]. AMPK is an upstream target of mTOR, and phosphorylated AMPK (p-AMPK) inhibits mTOR to alleviate podocytes senescence by regulating metabolism and inhibiting inflammation [5, 40]. Consistent with previous studies, our data showed that high glucose led to the down-regulation of p-AMPK and the up-regulation of p-mTOR and senescence genes (p16 and p53) in podocytes and renal tissues, suggesting that inhibition of autophagy aggravated high glucose-induced senescence through AMPK/mTOR pathway. Senescent podocytes could facilitate the senescence of neighboring cells through fibrinogenic senescence-associated secretory phenotype (SASP) paracrine signaling, resulting in diminished glomerular filtration rate and glomerulosclerosis [8, 35]. Notably, this study was the first to discover that AMPK/mTOR pathway-associated autophagy mediated the anti-senescence effects of hucMSCs. Moreover, the secretome of hucMSCs inhibited high glucose-induced podocytes senescence by activating autophagy, which was reversed by the autophagy inhibitor CQ, suggesting that paracrine molecules of hucMSCs activated autophagy to restore the podocyte senescence through AMPK/mTOR signaling pathway.

Currently, the treatment for DN is multiple, including antihyperglycemic drugs, lipid-lowering agents, renin–angiotensin–aldosterone system inhibitors (RAASi), and pharmacological alternatives for nephropathy [41, 42]. Sodium/glucose co-transporter 2 (SGLT-2) inhibitors have appeared as the first-line clinical drugs for DN treatment [43]. They can modulate hypoxia-inducible factors (HIF), e.g., suppression of HIF-1α and activation of HIF-2α, to improve oxidative stress in the kidney, but their renal protective and glucose-lowering effects seem less satisfactory in advanced DN patients [44, 45]. Previous meta-analyses of lipid-lowering agents showed a significant overall reduction in proteinuria and attenuation of interstitial inflammation and fibrosis in DN patients treated with statins, but no improvement was observed on glomerular filtration rate, serum creatinine and blood urea nitrogen levels [46]. RAASi treatment significantly reduced microalbuminuria by 37% at 24 weeks and restored normal albumin levels in urine at 28 weeks in DN patients, but neither glycated hemoglobin level nor mean creatinine clearance was restored over the course of RAASi treatment [14, 47, 48]. In this study, blood glucose level, 24 h urine volume, 24 h urinary protein, urine creatinine, and urinary albumin/creatinine ratio were effectively improved in diabetic rats in three weeks after hucMSCs injections. Morphologically, hucMSCs attenuated renal hypertrophy, glomerulosclerosis, and renal vacuolation in diabetic rats. These beneficial effects might be attributed to the homing capacity of MSCs, which allowed the injected MSCs to migrate to the injury site and thus repaired the damaged organ [49]. Besides, MSCs have been demonstrated to release paracrine substances, such as growth factors, cytokines, and exosomes, contributing to anti-inflammation and regeneration of renal tissues [21, 25]. Previous studies have also detected enhanced green fluorescent protein (eGFR)-labeled urine-derived stem cells in the kidneys of a diabetic rat model and a mouse model of ischemia–reperfusion injury, demonstrating that MSCs can exert regenerative effects by directly homing to damaged kidney tissue and can be transferred through the first-pass organ (lungs) [50, 51]. Similar results from our experiment echoed above findings. Our results indicated that most of the cells were localized in the lung tissues after 24 h of injection, but the majority of cells crossed the pulmonary circulation and reached the kidneys after 120 h of injection, and continued to rise at 168 h, indicating that the injection of hucMSCs through the tail vein could improve functional recovery. In contrast to the above results, reporter gene-marked hucMSCs were applied to rats through intravenous injections, and were imaged on days 1, 3, 5 post-injection. The data showed that there was a strong signal in the lungs by day 1 after injecting hucMSCs, which dropped by day 3 and disappeared by day 5. However, no signal of hucMSCs was observed in the organs in rats except for the lungs (Additional file 1: Fig. S4), owing to insufficient sensitivity of the experimental system. Therefore, mice or other animal systems might be more proper to study the distribution of reporter gene labeled MSCs. Our in vitro data showed that secretome from hucMSCs had positive regulative effects on senescence-related (*p16*, *p21*, *p53*) and autophagy-related (*Beclin-1*, *p62*, *LC3*) gene expressions in podocytes, indicating the paracrine-dependent mode of action of hucMSCs against podocytes senescence. Therefore, hucMSCs may be a promising stem cell therapy for DN. Although a significant reduction in blood glucose was observed after four weeks treatment of hucMSCs, the blood glucose level remained abnormally high. Some studies found that antidiabetics could not only lower the high blood glucose, but also benefit MSCs by enhancing their differentiation potential [52–54]. These findings indicate that combining hucMSCs with antidiabetics is an interesting and promising therapeutic strategy that may bring better outcomes for the treatment of DN. Further investigations are warranted to test the possibility of such combination strategy.

There are some limitations in this study. Firstly, rat renal podocytes were employed for in vitro assays, but the study on only podocytes was not adequate to cover all pathologies of DN. Other kinds of renal cells should be applied in future studies. Regrettably, human podocytes were not used for in vitro assays, which could bring higher translational impact to the study. In future, clinical trials should be conducted to determine the anti-DN effects of hucMSCs on patients. Secondly, autophagy is a complex process and this study still lacked in vivo evidence of the autophagy-related mechanism of hucMSCs. Further, the possibility of synergistic combination of MSCs with antidiabetics should be tested in future studies.

## Conclusion

This in vivo study demonstrated that hucMSCs could tend to accumulate at the kidney, decrease the blood glucose level, and ameliorate the renal dysfunction and histopathologic injury in diabetic rats. In vitro experiments showed that hucMSCs improved the cell viability, wound healing capacity, and senescent state of hyperglycemic-damaged podocytes mainly through paracrine action. The paracrine mechanism of hucMSCs was elucidated to be mediated by the AMPK/mTOR signaling pathway. Altogether, this study provides new insights of the renal protective roles of hucMSCs and the underlying paracrine-based actions, suggesting it was a promising stem cell-based therapy in the treatment of DN.

## Supplementary Information

**Additional file 1. Fig. S1:** Physical and biochemical analysis of rats. **Fig. S2:** Effects of different glucose concentrations on renal podocytes. **Fig. S3: **High glucose treatment inhibited damage repair ability of renal podocytes. **Fig. S4:** The distribution of hucMSCs with reporter gene in various organs in rats within 5 d.

**Additional file 2. Figs. S5–S29:** Full-length blots of three proteins, five proteins, nine proteins, eleven proteins, three proteinsof rat podocytes, four proteins of renal tissues.

**Additional file 3. Figs. S30–S34:** Full-length blots of four proteins of rat podocytes, and one protein of renal tissues exposed at different time points.

## Data Availability

All data generated or analyzed during this study are included in this published article or are available from the corresponding author on reasonable request.

## Abbreviations

DN: Diabetic nephropathy
MSCs: Mesenchymal stem cells
hucMSCs: Human umbilical cord-derived mesenchymal stem cells
MSC-CM: Conditioned medium of hucMSCs
STZ: Streptozotocin
FBG: Fasting blood glucose
DiD: 1,1’-Dioctadecyl-3,3,3’,3’-tetramethylindodicarbocyanine perchlorate
AMPK: Adenosine 5’-monophosphate (AMP)-activated protein kinase
mTOR: Mammalian target of rapamycin
