# Supplementary material for: Intravenous injection of human umbilical cord-derived mesenchymal stem cells ameliorates not only blood glucose but also nephrotic complication of diabetic rats through autophagy-mediated anti-senescent mechanism
Source: Stem Cell Res Ther. 2023 May 29;14:146. doi: 10.1186/s13287-023-03354-z (PMC10228071; doi:10.1186/s13287-023-03354-z)
Supplement: Supplementary file 1 — Additional file 1. Fig. S1: Physical and biochemical analysis of rats. Fig. S2: Effects of different glucose concentrations on renal podocytes. Fig. S3: High glucose treatment inhibited damage repair ability of renal podocytes. Fig. S4: The distribution of hucMSCs with reporter gene in various organs in rats within 5 d. [file 13287_2023_3354_MOESM1_ESM.pdf]

Supplementary File

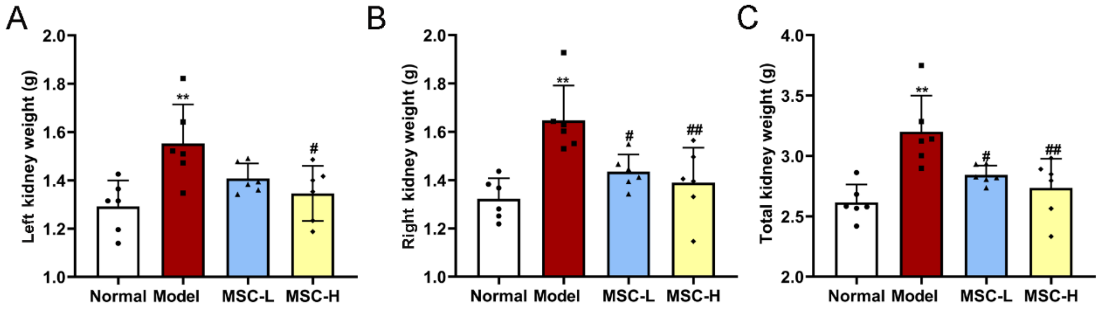

Figure S1. Physical and biochemical analysis of rats. Left kidney weight (A), right kidney weight (B), total kidney weight (C) in the normal (n = 6), model (n = 6), MSC-L (n = 6), and MSC-H (n = 6) groups. Data were shown as mean ± SD n=6, unpaired, student's *t*-test. *$P < 0.05$ and **$P < 0.01$ vs the normal group, $^{\#}P < 0.05$ and $^{\#\#}P < 0.01$ vs the model group.

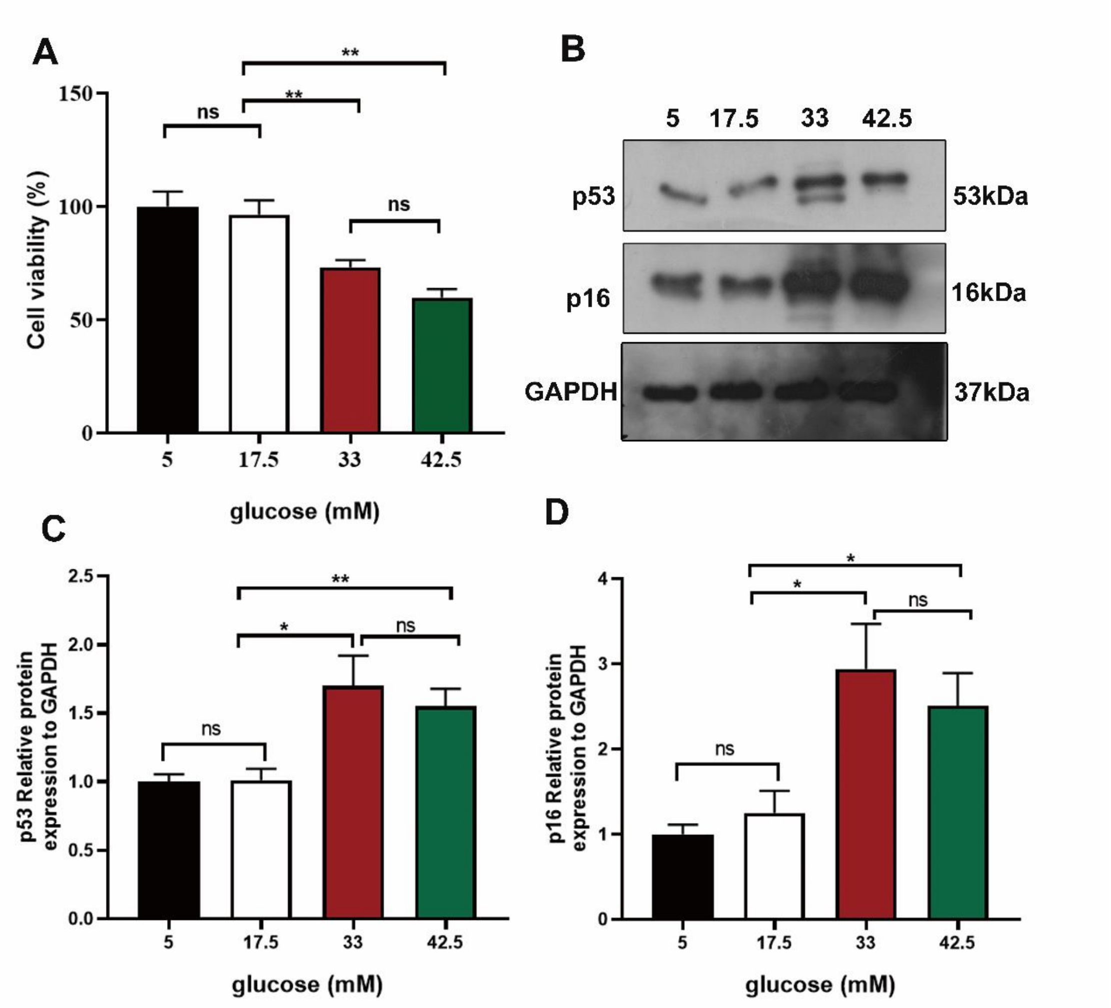

Figure S2. Effects of different glucose concentrations on renal podocytes. (A) CCK-8 assay for cell viability. (B) Representative western blot images of p53, p16, and GADPH in renal

podocytes. Full-length blots of rat podocytes were presented in Figure. S23-S25, respectively. (C) Histograms of statistical analysis on the levels of p53 and pl6 proteins in renal podocytes. Data were shown as mean ± SD, n = 3, unpaired, student's *t*-test. $^{**}P < 0.01$ vs the 17.5 mM group.

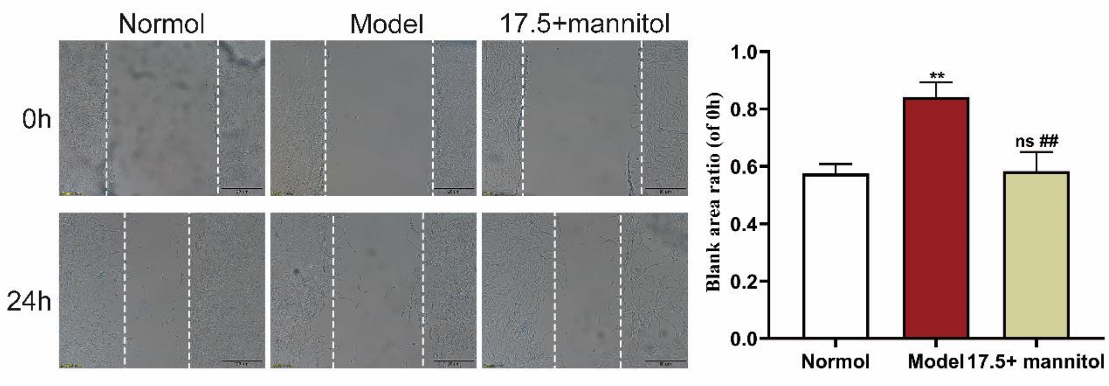

Figure S3. High glucose treatment inhibited damage repair ability of renal podocytes. Images at 0 and 24 h after scratches of each experimental condition and blank area radio (of 0 h) of each experimental condition. Data were shown as mean ± SD, n = 3, unpaired, student's *t*-test. $^{**}P < 0.01$ vs the normal group, $^{\#\#}P < 0.01$ vs the model group.

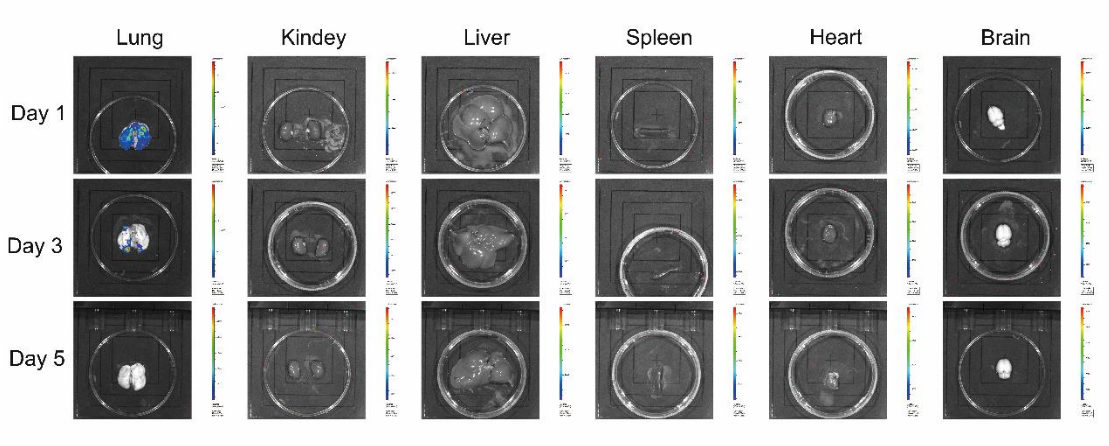

Figure S4. The distribution of hucMSCs with reporter gene in various organs in rats within 5 d.
