# Supplementary material for: Intravenous injection of human umbilical cord-derived mesenchymal stem cells ameliorates not only blood glucose but also nephrotic complication of diabetic rats through autophagy-mediated anti-senescent mechanism
Source: Stem Cell Res Ther. 2023 May 29;14:146. doi: 10.1186/s13287-023-03354-z (PMC10228071; doi:10.1186/s13287-023-03354-z)
Supplement: Supplementary file 2 — Additional file 2. Figs. S5–S29: Full-length blots of three proteins, five proteins, nine proteins, eleven proteins, three proteinsof rat podocytes, four proteins of renal tissues. [file 13287_2023_3354_MOESM2_ESM.pdf]

Except for those specifically indicated to be detected with the Gel Doc XR+ Imaging Systems (Bio-Rad, California, USA), the rest were developed with X-ray film.

The images appearing in the original manuscript have been marked with red boxes.

1. Full-length blots of three proteins (p16, p53, and GAPDH) of rat podocyte were presented in Figure. S5-S7.

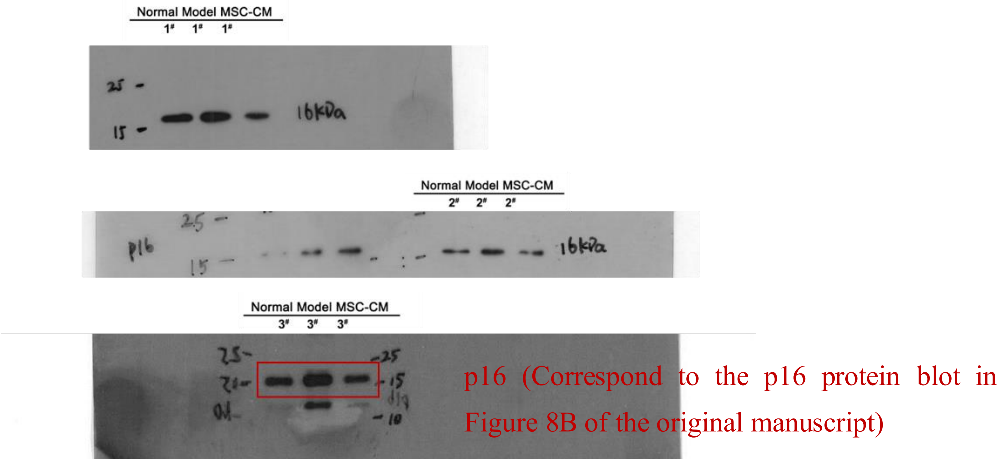

Figure. S5 Uncropped images of p16.

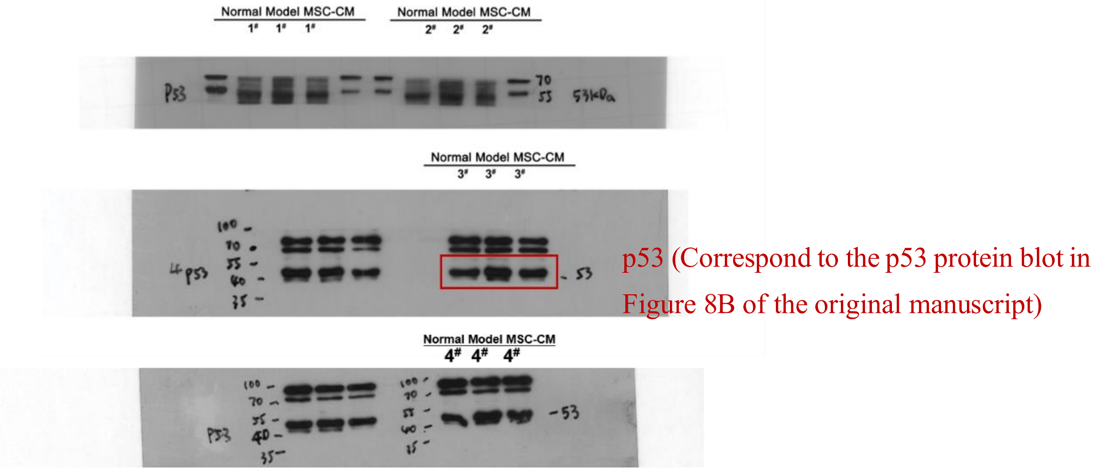

Figure. S6 Uncropped images of p53.

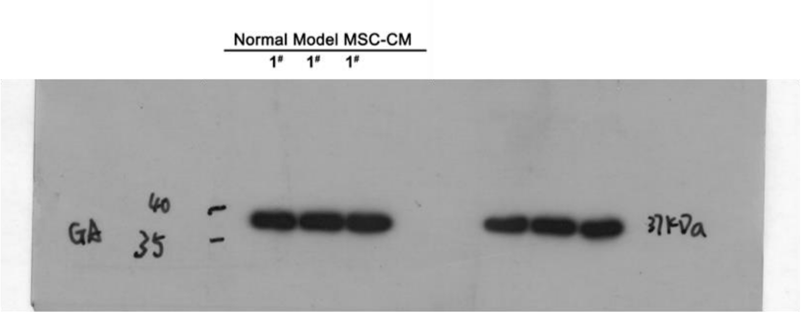

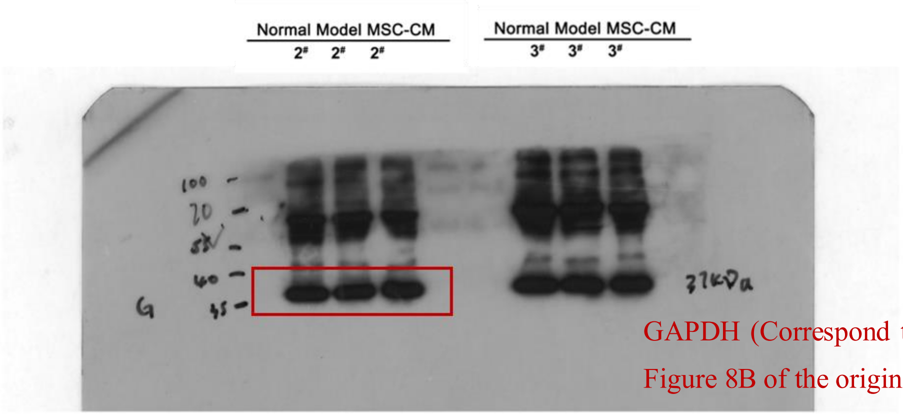

GAPDH (Correspond to the GAPDH protein blot in Figure 8B of the original manuscript)

Figure. S7 Uncropped images of GAPDH.

2. Full-length blots of five proteins (p-AMPK, p-mTOR, P16, P53, and GAPDH) of rat podocyte were presented in Figure. S8-S11. The bands were detected by Gel Doc XR+ Imaging Systems and displayed on X-ray film.

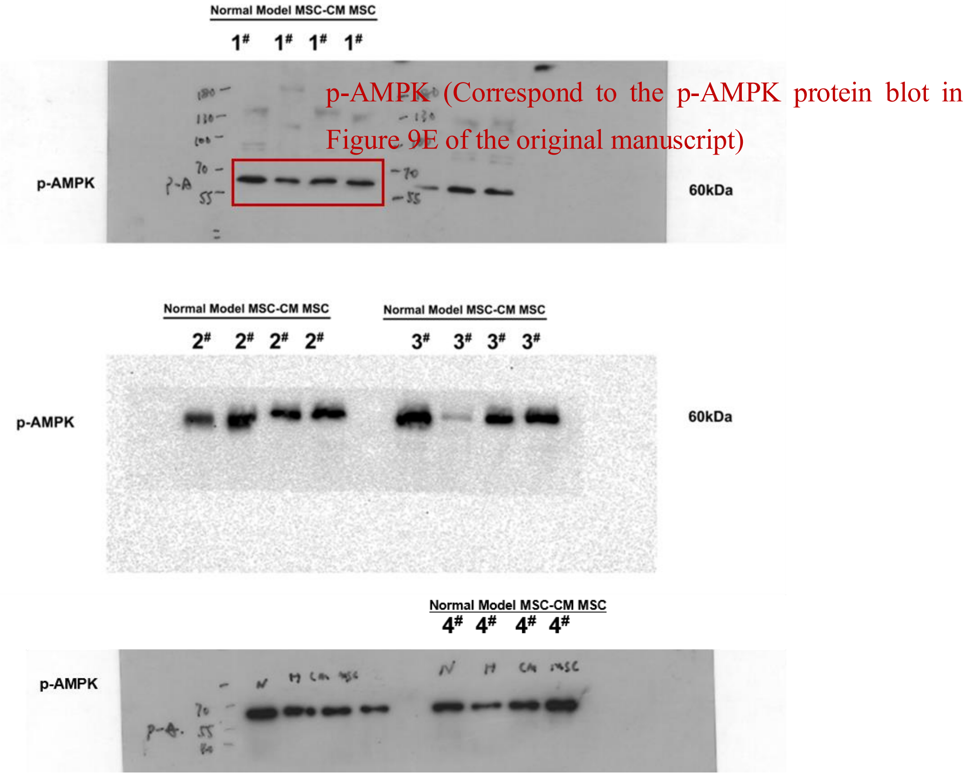

Figure. S8 Uncropped images of p-AMPK.

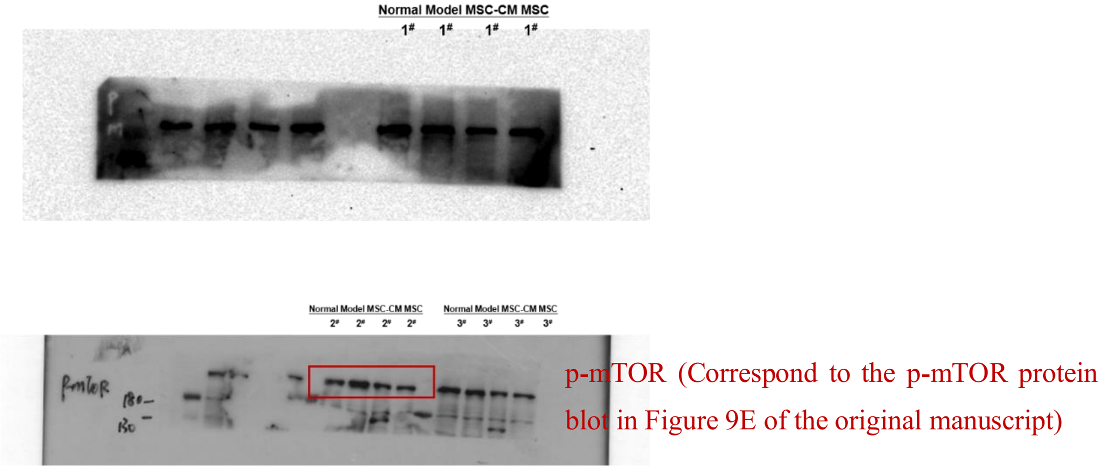

Figure. S9 Uncropped images of p-mTOR.

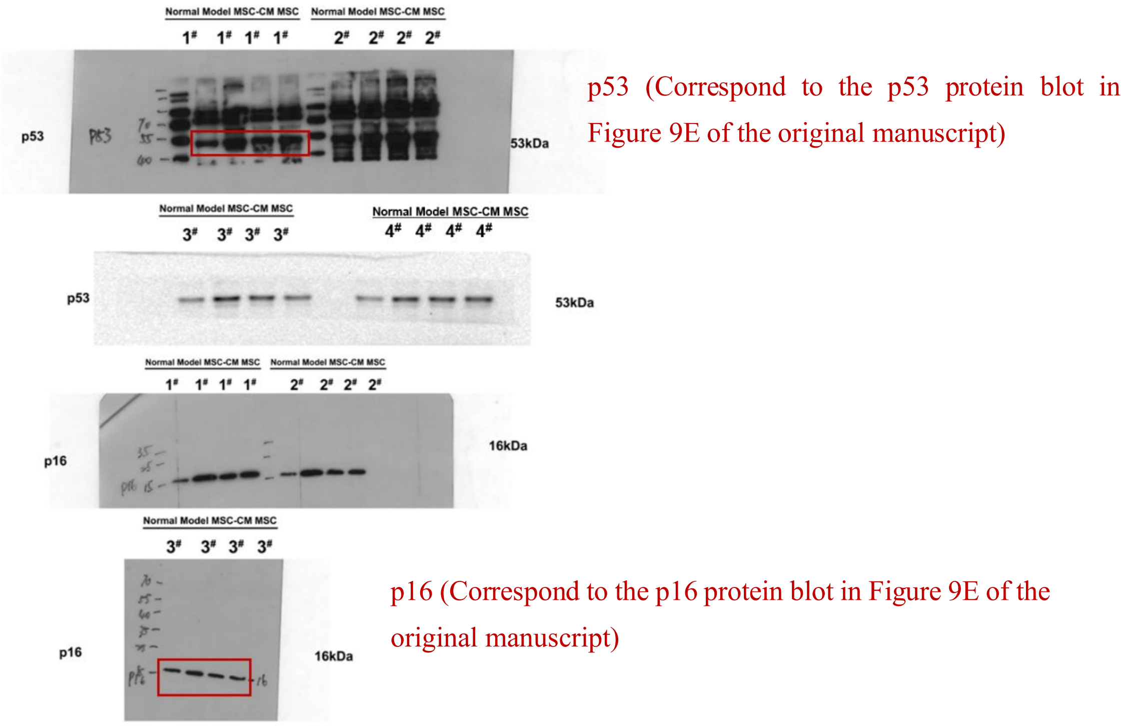

Figure. S10 Uncropped images of p53 and p16.

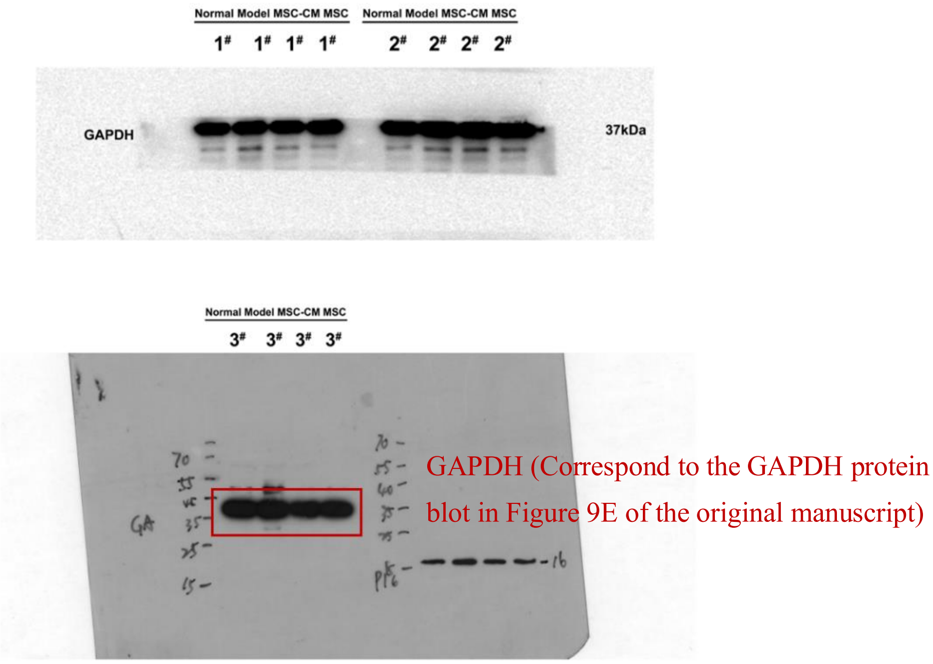

Figure. S11 Uncropped images of GAPDH.

3. Full-length blots of nine proteins (p62, Beclin-1, LC3, ULK1, AMPK, p-AMPK, mTOR, p-

mTOR, and GAPDH) of rat podocyte were presented in Figure.S12-S16.

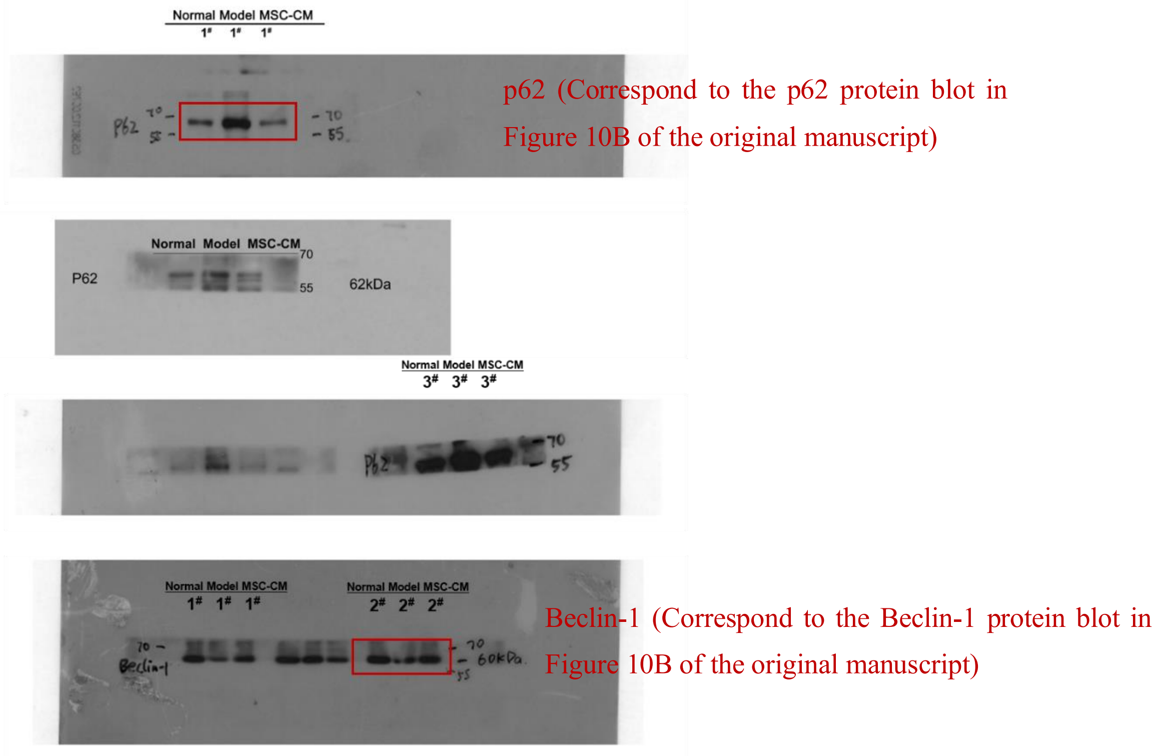

Figure. S12 Uncropped images of p62 and Beclin-1.

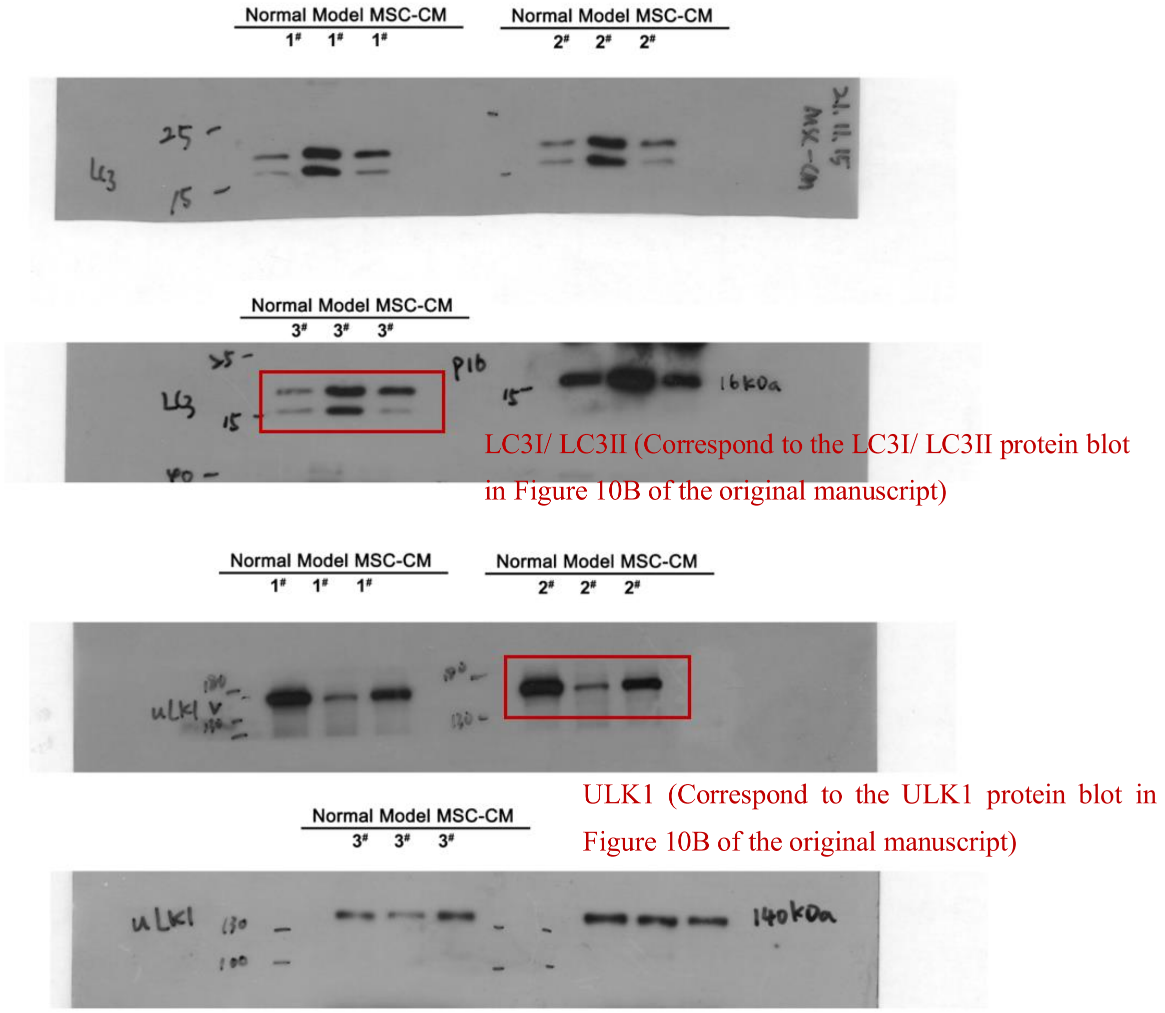

Figure. S13 Uncropped images of LC3 and ULK1.

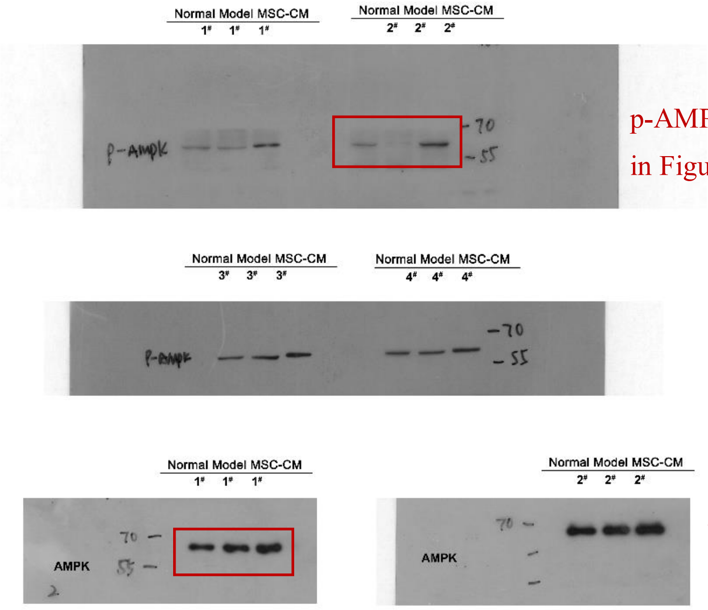

p-AMPK (Correspond to the p-AMPK protein blot in Figure 10C of the original manuscript)

AMPK (Correspond to the AMPK protein blot in Figure 10C of the original manuscript)

Figure. S14 Uncropped images of AMPK and p-AMPK.

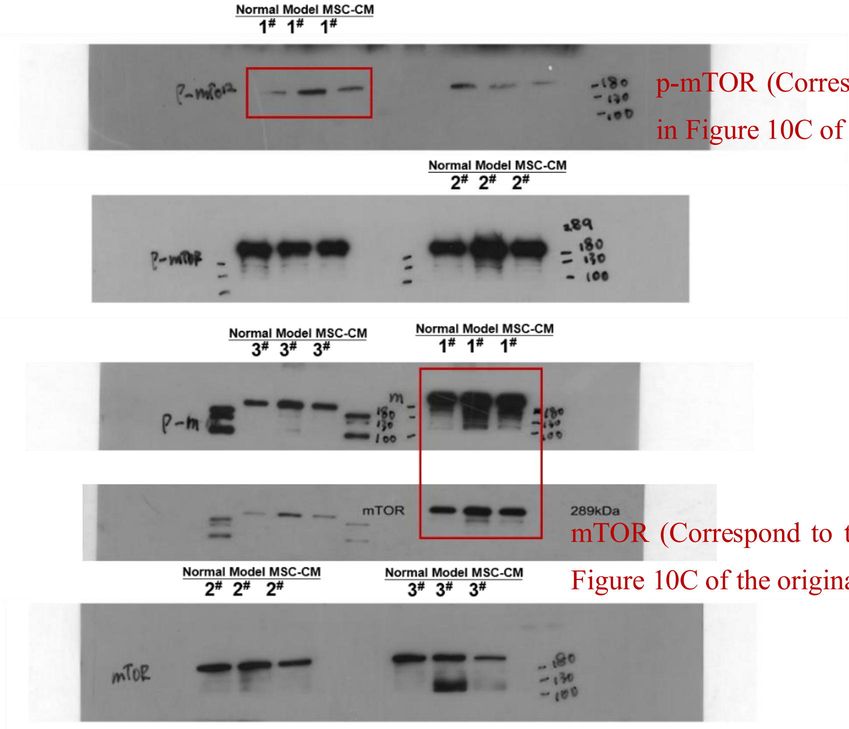

p-mTOR (Correspond to the p-mTOR protein blot in Figure 10C of the original manuscript)

mTOR (Correspond to the mTOR protein blot in Figure 10C of the original manuscript)

Figure. S15 Uncropped images of mTOR and p- mTOR. The reason for the above marks is that we expose them to multiple X-ray films during exposure.

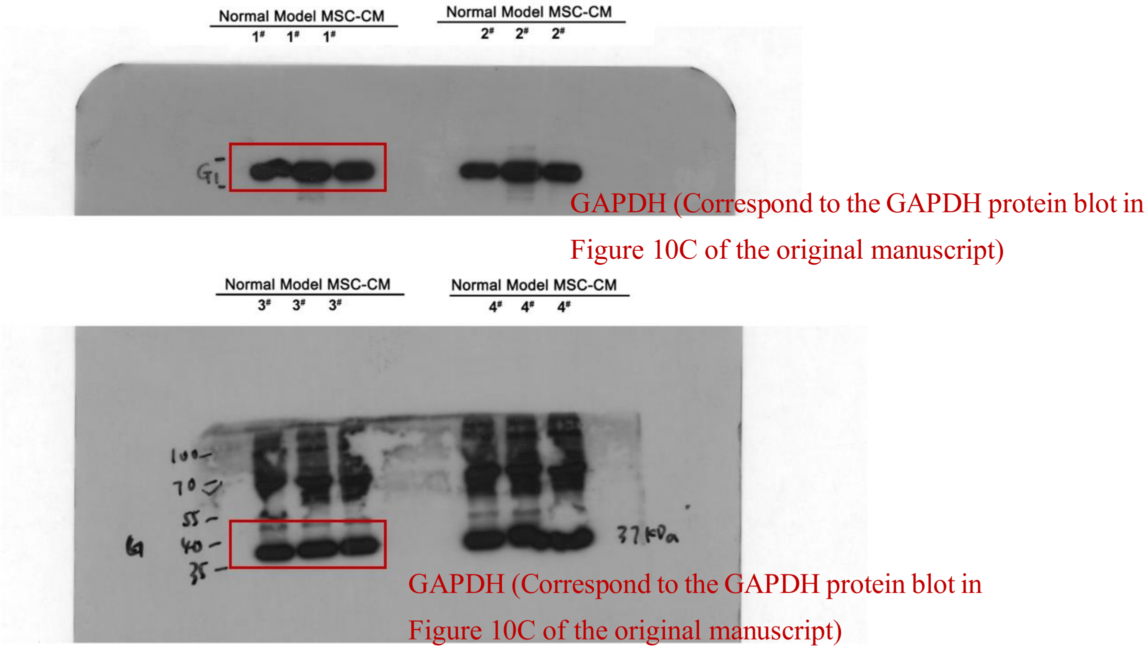

Figure. S16 Uncropped images of GAPDH.

4. Full-length blots of eleven proteins (AMPK, p-AMPK, mTOR, p-mTOR, p62, Beclin-1, LC3, ULK1, p16, p53 and GAPDH) of rat podocyte were presented in **Figure. S17-S22.**

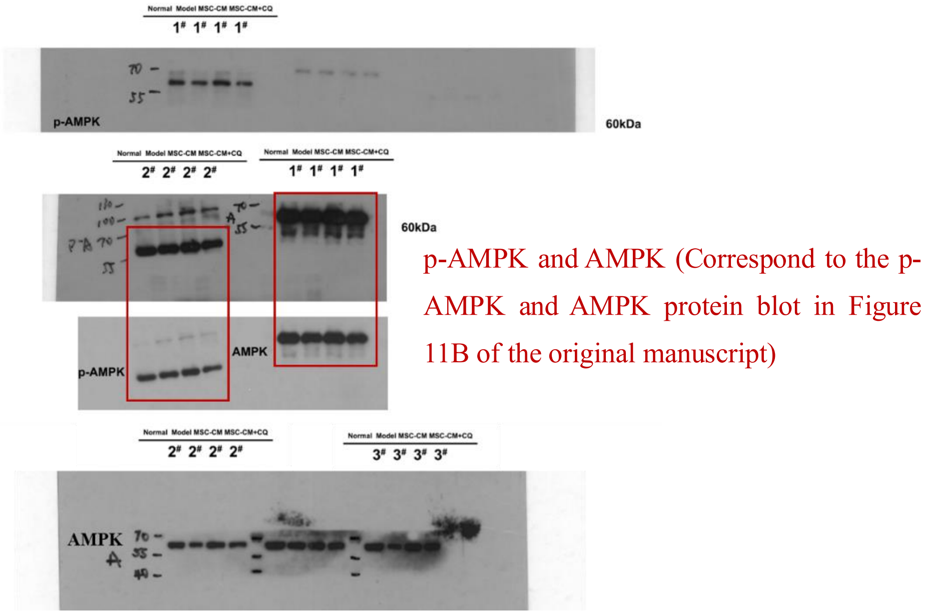

Figure. S17 Uncropped images of AMPK and p-AMPK. The reason for the above marks is that we expose them to multiple X-ray films during exposure.

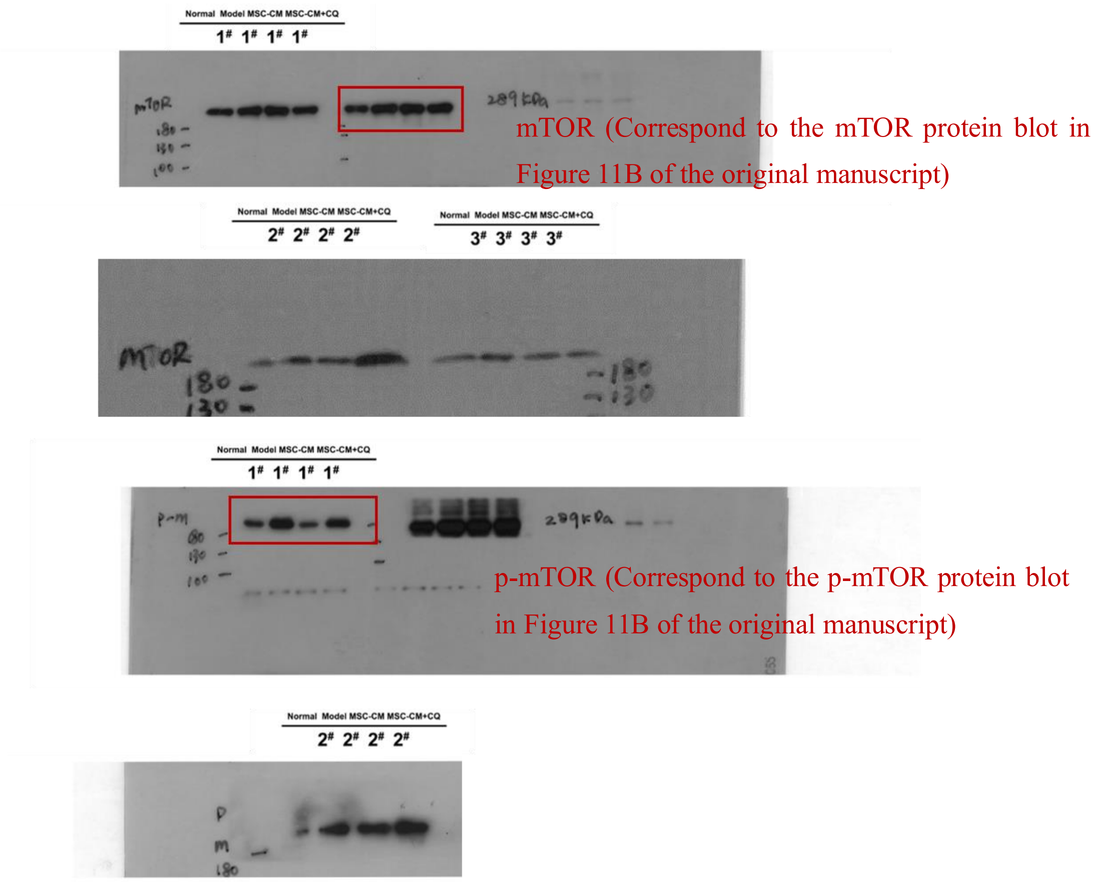

Figure. S18 Uncropped images of mTOR and p-mTOR.

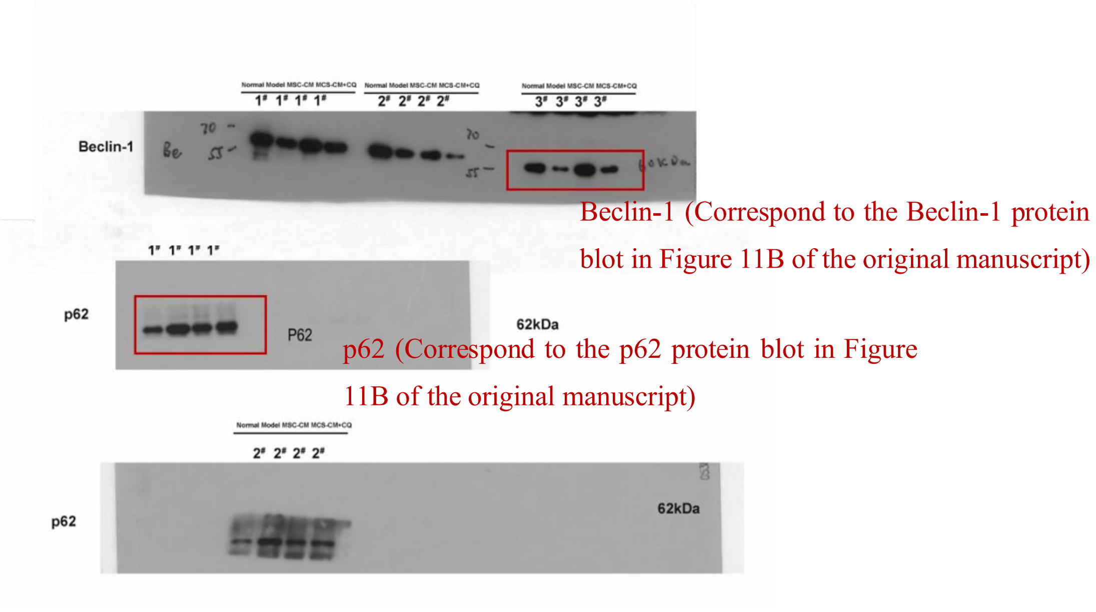

Figure. S19 Uncropped images of Beclin-1 and p62.

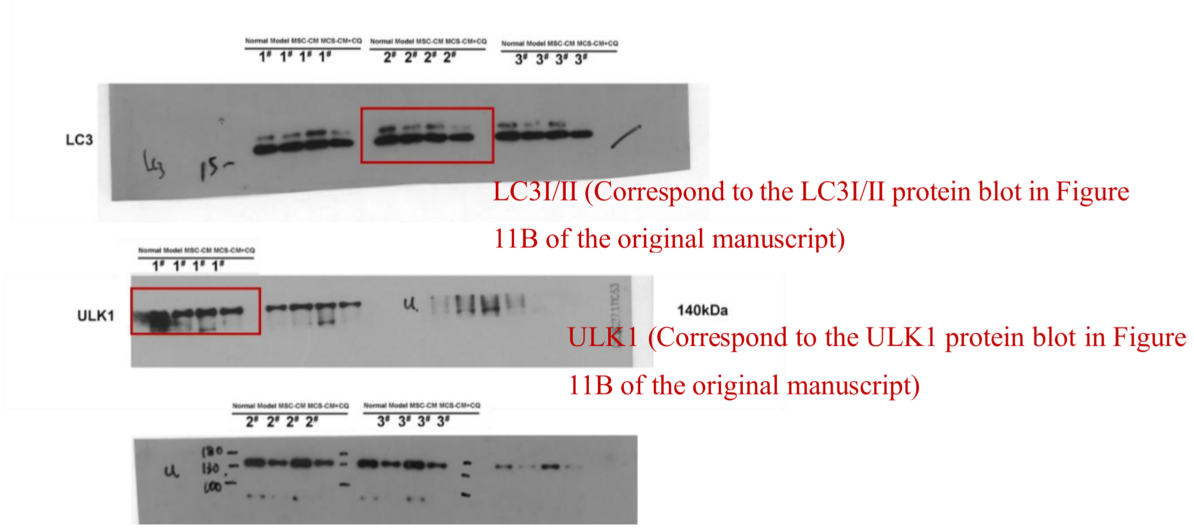

Figure. S20 Uncropped images of LC3 and ULK1.

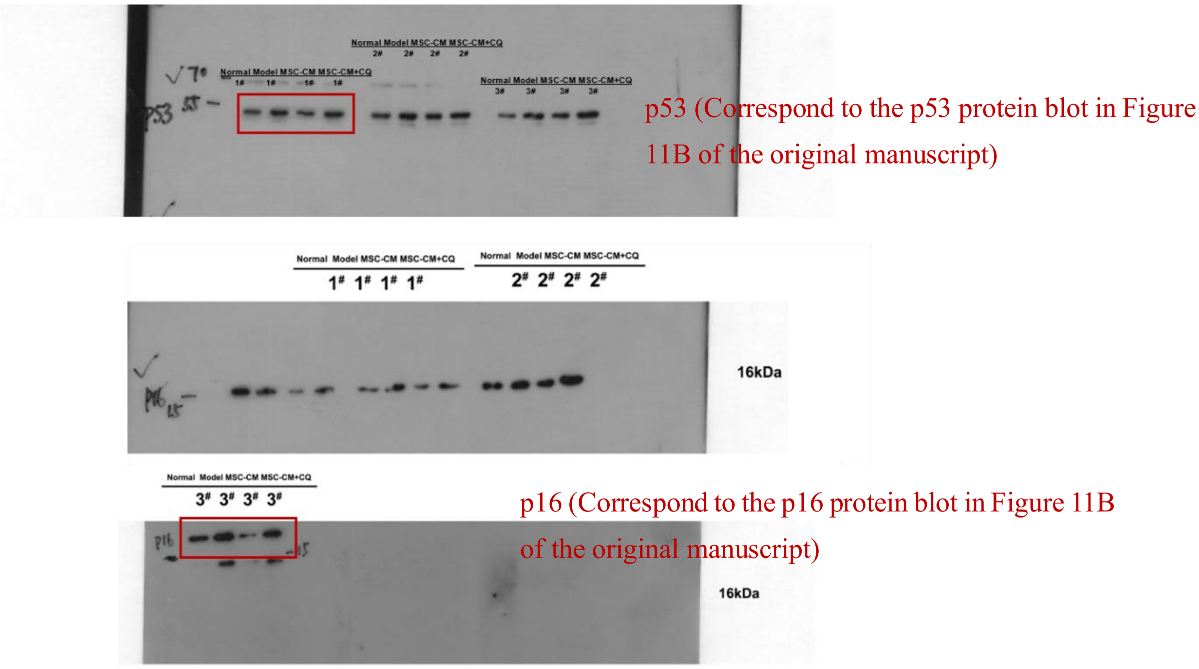

Figure. S21 Uncropped images of p53 and p16.

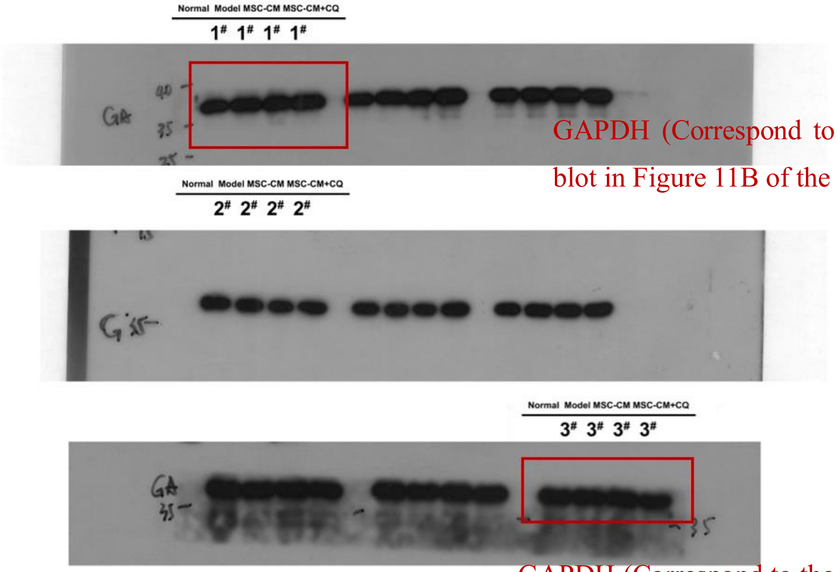

GAPDH (Correspond to the GAPDH protein blot in Figure 11B of the original manuscript)

GAPDH (Correspond to the GAPDH protein blot in Figure 11B of the original manuscript)

Figure. S22 Uncropped images of GAPDH.

5. Full-length blots of three proteins (p16, p53 and GAPDH) of rat podocyte were presented in Figure. S23-S25.

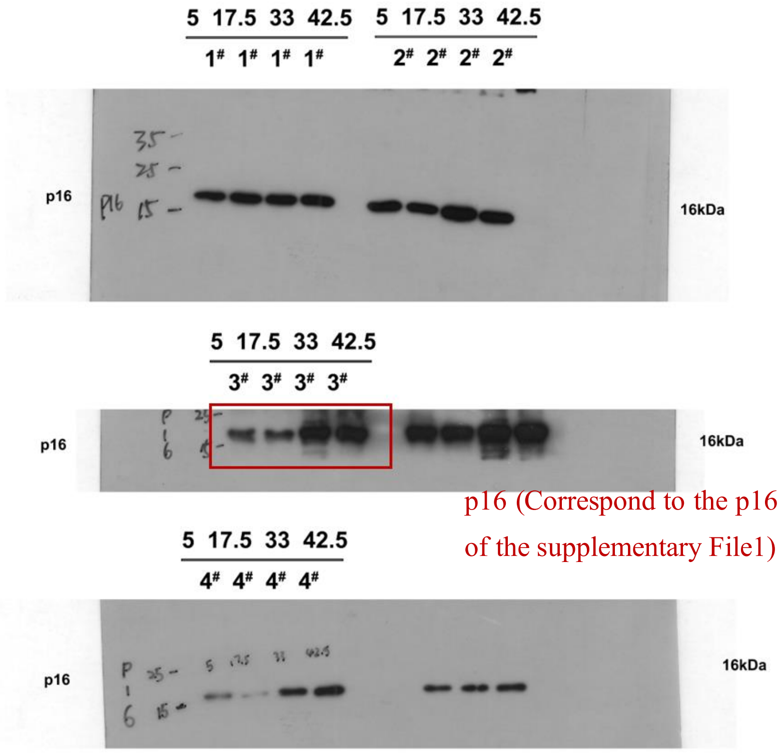

p16 (Correspond to the p16 protein blot in Figure S2B of the supplementary File1)

Figure. S23 Uncropped images of p16.

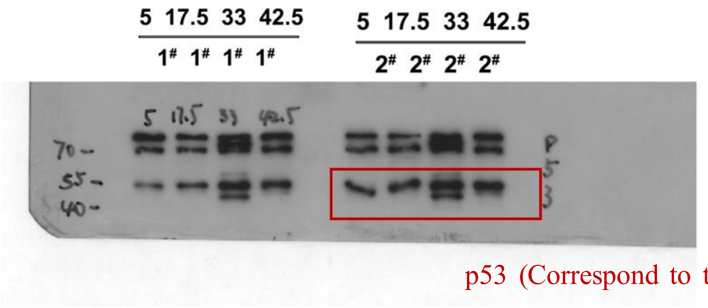

p53 (Correspond to the p53 protein blot in Figure S2B of the supplementary File1)

Figure. S24 Uncropped images of p53.

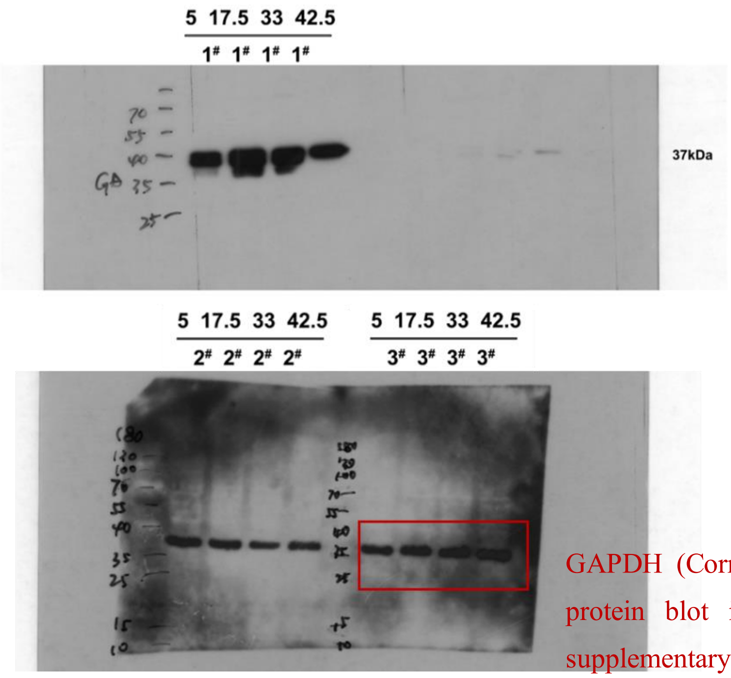

GAPDH (Correspond to the GAPDH protein blot in Figure S2B of the supplementary File1)

Figure. S25 Uncropped images of GAPDH.

6. Full-length blots of four proteins (p-AMPK, p-mTOR, p16, and GAPDH) of renal tissue were presented in Figure. S26-S29.

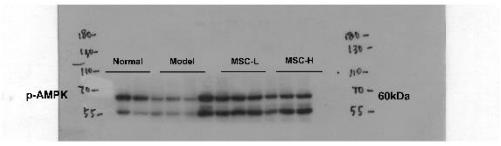

Figure. S26 Uncropped images of p-AMPK. Correspond to the p-AMPK protein blot in Figure 5C of the original manuscript.

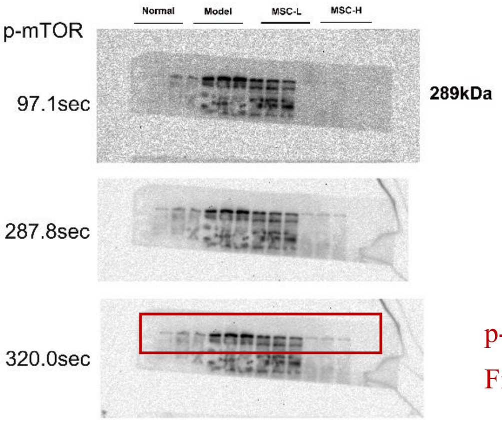

p-mTOR (Correspond to the p-mTOR protein blot in Figure 5C of the original manuscript)

Figure. S27 Uncropped images of p-mTOR. The band detected by Gel Doc XR+ Imaging Systems.

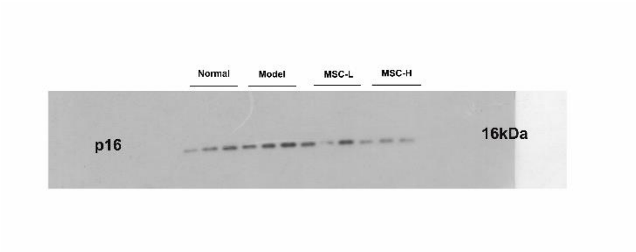

Figure. S28 Uncropped images of p16. Correspond to the p-AMPK protein blot in Figure 5C of the original manuscript.

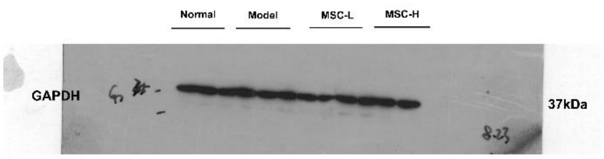

Figure. S29 Uncropped images of GAPDH. Correspond to the p-AMPK protein blot in Figure 5C of the original manuscript.
