# Supplementary material for: Intravenous injection of human umbilical cord-derived mesenchymal stem cells ameliorates not only blood glucose but also nephrotic complication of diabetic rats through autophagy-mediated anti-senescent mechanism
Source: Stem Cell Res Ther. 2023 May 29;14:146. doi: 10.1186/s13287-023-03354-z (PMC10228071; doi:10.1186/s13287-023-03354-z)
Supplement: Supplementary file 3 — Additional file 3. Figs. S30–S34: Full-length blots of four proteins of rat podocytes, and one protein of renal tissues exposed at different time points. [file 13287_2023_3354_MOESM3_ESM.pdf]

Images exposed at different time points by using GelDoc XR+imaging system.

Full-length blots of five proteins (p-AMPK, p-mTOR, P16, P53, and GAPDH) of rat podocyte were presented in Figure. S30-33. The same band detected by GelDoc XR+imaging system at different exposure time points.

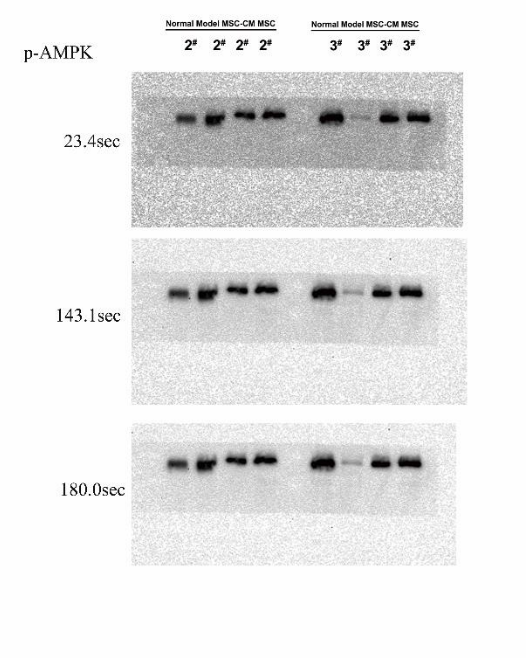

Figure. S30 Uncropped images of p-AMPK exposed at different time points .

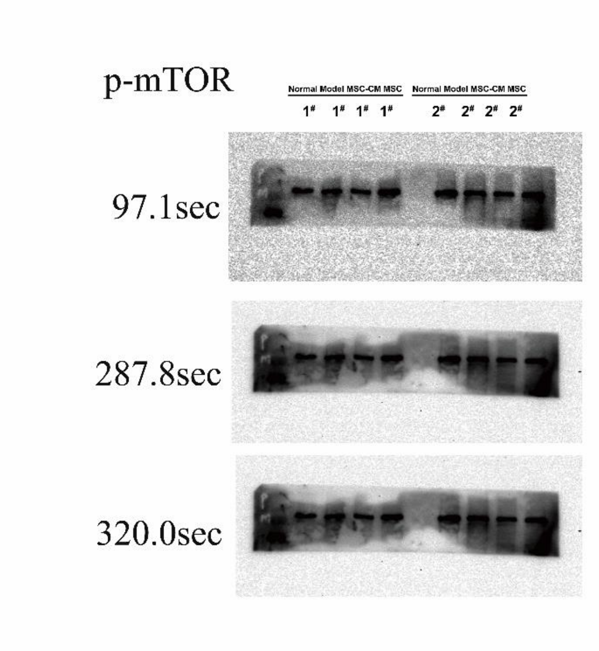

Figure. S31 Uncropped images of p-mTOR exposed at different time points.

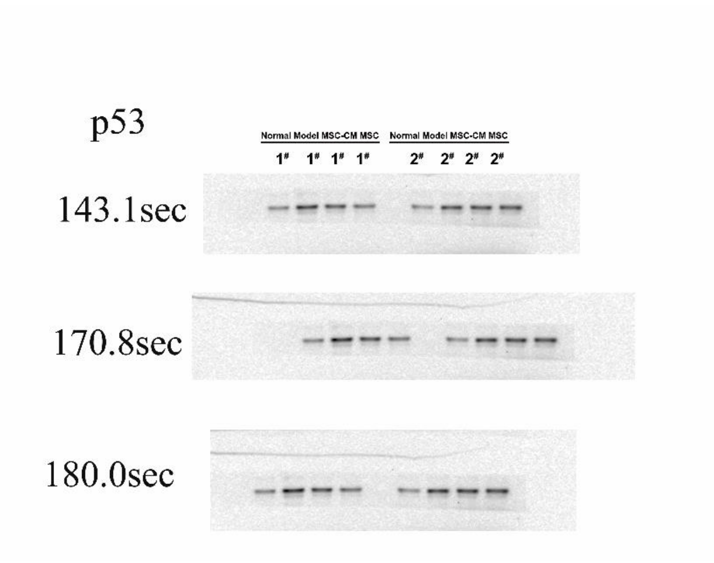

Figure. S32 Uncropped images of p53 exposed at different time points

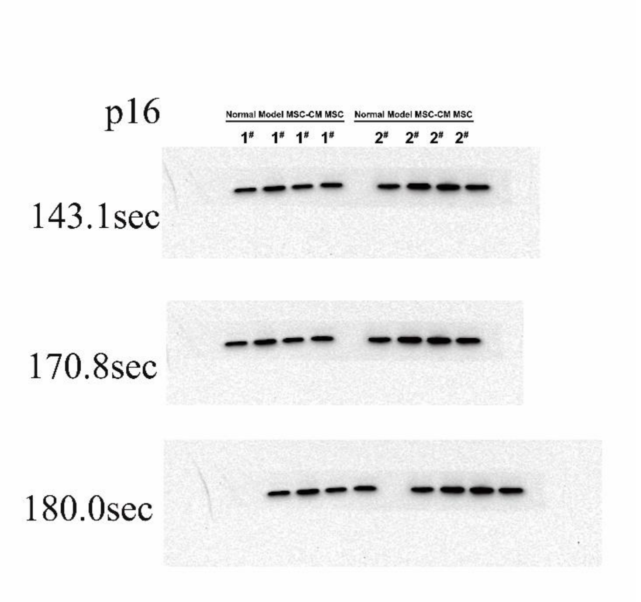

Figure. S33 Uncropped images of p16 exposed at different time points.

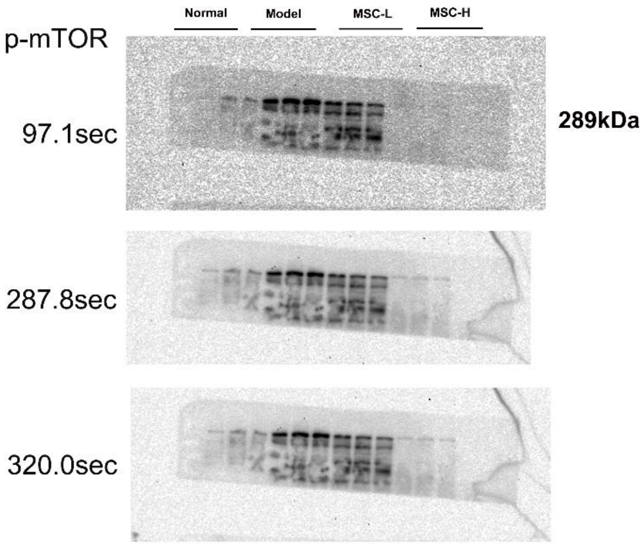

Figure. S34 Uncropped images of p-mTOR. The band detected by Gel Doc XR+ Imaging Systems at different exposure time points.
